# The effects of magnesium and vitamin D/E co-supplementation on inflammation markers and lipid metabolism of obese/overweight population: a systematic review and meta-analysis

**DOI:** 10.3389/fnut.2025.1563604

**Published:** 2025-09-01

**Authors:** Kai Deng, Jiamei Liu, Ye Miao, Guanqi Wang, Xingli Wang, Shengye Liu, Liyu Yang

**Affiliations:** ^1^Department of Urology, The First Hospital of China Medical University, Shenyang, China; ^2^Department of Pathology, The Shengjing Hospital of China Medical University, Shenyang, Liaoning, China; ^3^Department of Laboratory Medicine, The Shengjing Hospital of China Medical University, Shenyang, Liaoning, China; ^4^Department of Rehabilitation, Milian Community Health Service Center, Shenyang, Liaoning Province, China; ^5^Department of Ophthalmology, The Fourth People's Hospital of Shenyang, Shenyang, Liaoning, China; ^6^Department of Orthopedics, The Shengjing Hospital of China Medical University, Shenyang, Liaoning, China

**Keywords:** magnesium, vitamin D, vitamin E, inflammation, meta-analysis

## Abstract

**Background:**

Inflammatory reactions can induce or facilitate the occurrence and development of various diseases in the human body. It is crucial to regulate and actively control inflammatory factors to maintain the health of an individual. Vitamins D and E and magnesium ions may potentially inhibit inflammatory responses. Abnormal lipid metabolism is known to affect people’s health and lead to serious diseases. Magnesium and vitamin E are also known to possess anti-lipidemic properties. It is worth noting that the prevalence and disease burden of some diseases are related to overweight and obesity. This systematic review and meta-analysis assesses the impact of magnesium and vitamin D or vitamin E co-supplementation on inflammation and lipid metabolism markers of obese/overweight population in randomized controlled trials (RCTs).

**Methods:**

A comprehensive search was conducted across PubMed, Web of Science, Embase and Cochrane databases until January 2024 to investigate the impact of simultaneous supplementation of magnesium and vitamin D/E. In both intervention and control groups, the research analyzed the pooled mean difference (MD) and the associated 95% confidence interval (CI) of marker levels of inflammation and lipid metabolism.

**Results:**

Meta-analysis of nine RCTs (total of 509 individuals) showed that magnesium and vitamin D significantly elevated the levels of 25(OH)D (MD:13.37, 95%CI: 0.45, 26.29, *p* = 0.04) and magnesium (MD: 0.21, 95% CI: 0.16, 0.27, *p* < 0.00001). Co-supplementation of magnesium and vitamin D/E lowered levels of serum hypersensitivity C-reactive protein (hs-CRP) (MD: −1.19, 95%CI: −1.95, −0.42, *p* = 0.002). In subgroup analysis, serum levels of hs-CRP was notably reduced in individuals receiving magnesium and vitamin D supplementation (MD = −0.66, 95%CI: −1.17, −0.14, *p* = 0.01). However, no significant differences were observed between magnesium and vitamin E supplementation (MD: −3.54, 95%CI: −9.52, 2.43, *p* = 0.25). The combination of magnesium and vitamin D significantly reduced tumor necrosis factor-*α* (TNF-α) levels (MD: −0.87, 95%CI: −1.62, −0.11, *p* = 0.02). In contrast, the serum levels of interleukin-6 (IL-6) showed a non-significant decrease (MD: −0.09, 95%CI: −0.33, 0.15, *p* = 0.46), and did not significantly affect lipid metabolism according to levels of parameters such as serum triglyceride (MD = 1.84, 95% CI: −28.92, 32.60, *p* = 0.91), serum LDL-c (MD: −4.56, 95% CI: −14.19, 5.08, *p* = 0.35), and serum HDL-c (MD: 1.96, 95% CI: −3.07, 6.98, *p* = 0.45) in the co-supplementation of magnesium and Vitamin E.

**Conclusion:**

This study demonstrates a notable decrease in hs-CRP and TNF-*α* levels through vitamin D and magnesium co-supplementation in individuals. Particularly, middle-aged women with vitamin D deficiency, and obese or overweight participants, may experience specific benefits from vitamin D and magnesium co-supplementation in reducing inflammatory response. However, magnesium and vitamin E supplementation did not significantly reduce the indicators of lipid metabolism.

**Systematic review registration:**

https://www.crd.york.ac.uk/prospero/#loginpage

## 1 Introduction

Inflammation, a non-specific response of the body to injury, infection, or irritation, typically involves processes such as vasodilation, white blood cell migration, and tissue repair. The inflammatory response protects the body from harm by activating immune system and releasing inflammatory mediators, and it is a common feature of many diseases like osteoarthritis, dermatopathy, and lung infections, etc. (1). Lipids have important roles in energy metabolism; perturbations of lipid metabolism are responsible for the development of various pathologies, including metabolic syndrome, obesity, and type 2 diabetes (2). Obesity is an important risk factor contributing to the burden of disease worldwide. Body weight is influenced by the interaction of genetic, environmental and psychosocial factors that act through several physiological mediators of food intake and energy expenditure that affect fat deposition (3).

Vitamin D is a lipid-soluble vitamin, and 1,25-dihydroxycholecalciferolis its active form. While this vitamin can be obtained from food, the majority is synthesized in the skin. Vitamin D undergoes metabolism in the body to exert its effects through its active metabolites (4). It primarily plays an indispensable role in maintaining calcium and phosphate homeostasis, as well as bone density within the body (5). Vitamin D exhibits various pharmacological effects, including anti-inflammatory (6), and immune-modulatory properties (7). Recent meta-analyses have highlighted their significant impact on inflammatory markers and lipid profiles, particularly in the context of chronic inflammatory diseases and metabolic disorders. Specifically, studies have shown that higher levels of vitamin D are inversely associated with systemic inflammation markers, such as C-reactive protein (CRP) and interleukin-6 (IL-6) (8). Similarly, magnesium intake has been linked to improved lipid parameters, including reductions in low-density lipoprotein cholesterol (LDL-C) and triglycerides, which are critical for cardiovascular health (9). These findings underscore the importance of these micronutrients in managing chronic diseases and maintaining overall health. The relationship between vitamin D and inflammation has garnered considerable attention, particularly in light of its potential role in modulating immune responses. Vitamin D deficiency has been associated with heightened inflammation, which is a contributing factor in various chronic diseases, including cardiovascular disease and diabetes (10). Moreover, Vitamin E also modulates T cell function by directly impacting the integrity of T cell membrane, signal transduction, and cell division, and also indirectly by affecting inflammatory mediators generated from other immune cells (11). Studies indicate that vitamin E exerts various potentially beneficial effects on human health, such as anti-lipidemic (12, 13), anti-inflammatory (14), anti-obesity (15).

Magnesium is an essential element for maintaining normal life metabolism in organisms (16). It is one of the primary cations in human intracellular fluid, with levels second only to potassium ions. Magnesium and inflammatory factors exert a mutual regulatory relationship (17). Clinical studies have shown that insufficient intake of magnesium can cause peripheral vascular congestion in the body (18), resulting in degranulation of mast cells and the secretion of large amounts of histamine and inflammatory mediators (19). Mg^2+^ deficit and its lower blood concentration is frequent in patients with the main risk factors, hyperlipidemia, hypertension, diabetes, and obesity. Magnesium can help lower triglyceride (TG) levels and raise HDL-cholesterol levels (20). Magnesium is a critical cofactor for enzymes involved in vitamin D metabolism, including 25-hydroxylase and 1α-hydroxylase, which convert vitamin D to its active form (21). Magnesium deficiency may impair vitamin D activation, thereby reducing its anti-inflammatory efficacy. Conversely, adequate magnesium levels enhance vitamin D bioavailability, potentially amplifying its inhibitory effects on pro-inflammatory cytokines such as TNF-*α* (22). Vitamin E mitigates oxidative stress by neutralizing free radicals, while magnesium suppresses NF-κB signaling to reduce inflammation (23). Their co-supplementation may synergistically attenuate oxidative damage and inflammatory cascade, yet clinical evidence remains sparse and conflicting. There is growing interest in using a strategy that combines both magnesium and vitamins, as this may enhance metabolic profiles in various diseases characterizing metabolic irregularities. Research has suggested a potential association between vitamin D and magnesium (24). In addition, due to their unique anti-inflammatory properties, combining vitamin D and magnesium supplements may improve anti-inflammatory effects, further lowering the production of inflammatory compounds and regulating metabolism (25). On the other hand, the studies on magnesium and vitamin E co-supplementation may have conflicting conclusions on certain research indicators, and the sample sizes are small, making effective conclusions difficult. Although vitamin D and vitamin E exhibits antioxidant properties and magnesium influences lipid metabolism, no systematic review has comprehensively evaluated their co-administration. This study addresses this critical gap by synthesizing evidence from randomized controlled trials (RCTs) to assess the combined effects of magnesium with vitamins D/E on inflammation and lipid metabolism in overweight/obese populations—a high-risk group for metabolic and inflammatory comorbidities.

## 2 Materials and methods

The current meta-analysis was conducted as per the guidelines outlined in the PRISMA checklist (26) for Systematic Reviews and Meta-analysis. The research protocol has been properly registered on PROSPERO (CRD42024498691).

### 2.1 Search strategy and eligibility criteria

We searched the Cochrane Library, Embase, PubMed, and Web of Science from their founding dates until January 2024, for this investigation. The quick search terms comprised (Vitamin D OR Vitamin E) AND (Magnesium) AND (Co-supplementation) AND (Inflammation OR metabolism OR Oxidative stress). The advanced retrieval formulas were displayed in Supplementary Table 1. Following the elimination of duplicate articles, two reviewers (Deng K and Yang Ly) independently examined the initially retrieved literature using the below-specified inclusion and exclusion criteria. The inclusion criteria comprised studies specifically comparing the effectiveness of co-supplementation of magnesium and vitamin D/E with placebo supplementation lacking magnesium and vitamin D/E, and the intended outcomes were documented. On the other hand, the exclusion criteria were studies that were not RCTs, those that explored patients receiving treatment for purposes unrelated to of magnesium and vitamin D/E co-supplementation, and studies for which the full text was inaccessible.

### 2.2 Data extraction

For the meta-analysis, two independent reviewers (Deng K and Yang Ly) were tasked with data extraction from qualifying studies. The extracted data included the primary author, year of publication, patient demographics, intervention and placebo group sizes, intervention specifics (Mg + vitamin D/E dosage), post-intervention group differences (Mg + vitamin D/E vs. placebo), and follow-up duration. The examined outcomes comprised serum levels of 25-hydroxyvitamin D(25(OH)D; ng/mL), magnesium (Mg; mg/dL), high sensitivity C-reactive protein (hs-CRP; mg/L), interleukin-6 (IL-6; pg./mL), tumor necrosis factor-*α* (TNF-α; pg./mL), triglycerides (TG; mg/dL), low density lipoprotein cholesterol (LDL-c; mg/dL), and high density lipoprotein cholesterol (HDL-c; mg/dL).

### 2.3 Risk of bias assessment

To assess the risk of bias, the Cochrane tool for evaluating bias risk (27) was utilized. Areas reviewed included generation of random sequences (selection bias), concealment of allocation (selection bias), masking of participants and staff (performance bias), masking of outcome evaluation (bias in detection), incomplete outcome data (attrition bias), selective reporting (bias in reporting), and miscellaneous bias factors. Ratings of ‘+’ denote low risk of bias, ‘?’ suggest uncertainty, and ‘−’ indicate high risk.

### 2.4 Statistical analysis

In each investigation, data obtained from the co-supplementation group and the control group post-intervention were utilized. Mean and standard deviation (SD) were used to represent continuous variables. Kheyruri’s study (28) presented 25(OH)D levels, serum magnesium levels, hs-CRP levels, and TNF-*α* levels as median (interquartile). Mean and SD were estimated according to Luo et al. (29) and Wan et al. (30) respectively. Their respective 95% confidence intervals (95%CI) were summarized for forest plots. Study heterogeneity was visually represented through forest plots, and statistical assessment was conducted using Cochran’s Q test and the I^2^ index. An I^2^ value >50% indicated notable heterogeneity, prompting the utilization of a random effects model, while an I^2^ value of 50% or less led to the application of a fixed effects model. When feasible, sensitivity analysis was performed to address substantial heterogeneity. Statistical significance was determined by a *p*-value <0.05 in the overall effect test. Data were pooled utilizing the meta-analytic approach within Cochrane Review Manager 5.4.1 and STATA 18. The assessment of publication bias was conducted by visually inspecting funnel plots and utilizing Egger’s test. Subgroup analyses were conducted based on the study characteristics (such as type of supplement, supplementary dosage) for outcome measures exhibiting considerable heterogeneity, to identify possible sources of this variability. Furthermore, sensitivity analysis was conducted to assess the stability of the study findings.

## 3 Results

### 3.1 Search results

**Figure 1:**
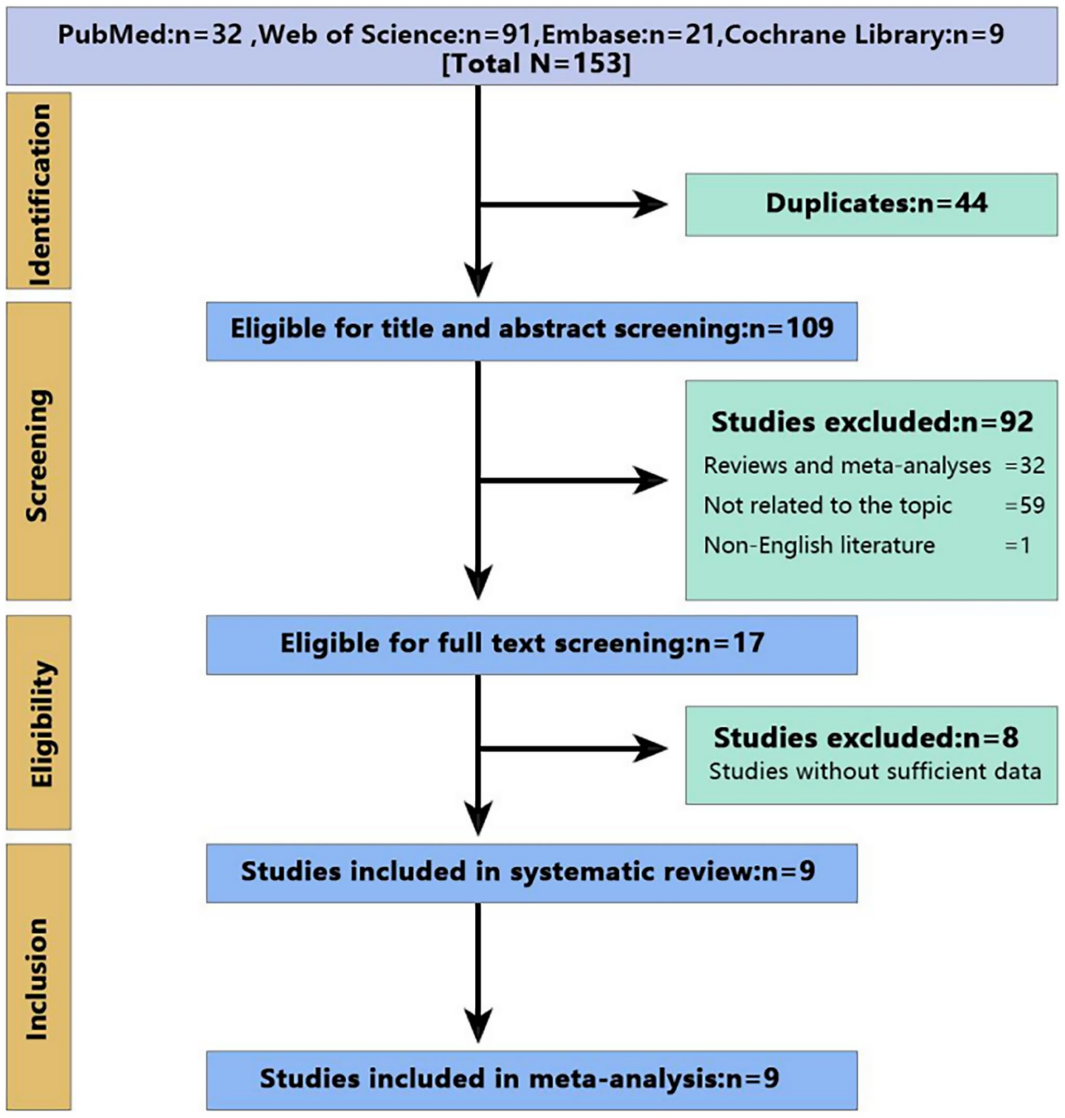
Flow diagram of PRISMA study selection.

Figure 1 visually represents the study selection process. Out of the 153 studies reviewed individually, full text of 17 was screened. During this screening stage, eight studies lacking adequate data were eliminated, and ultimately, nine studies (25, 28, 31–37), encompassing a collective sample size of 509 patients, were chosen for the meta-analyses.

### 3.2 Study characteristics

General information about the included studies is presented in Table 1. In this article, nine studies were included, wherein a total of 109 participants were administered a combination of magnesium and vitamin D supplementation, while 119 participants were given a placebo as a control. There were a total of 136 participants in both the intervention group of magnesium and vitamin E and the placebo group. Among obese/overweight population, four studies (25, 28, 31, 32) investigated the effects of Mg and vitamin D co-supplementation on inflammatory markers while five studies (33–37) focused on the effects of Mg and vitamin E co-supplementation.

**Table 1:** Characteristic of studies included in the meta-analyses.

First author et al., year	Age (years, mean ± SD or range)	Sex	Number of participants (intervention/placebo)	Health conditions	Intervention	Outcome (Difference between groups at the end of the intervention)	Follow-up
Farvid et al,. 2004 (33)	50.6 ± 9.7 (intervention) 49.6 ± 9.2 (placebo)	F/M	17/18	Type 2 diabetes	100 mg Mg +15 mg Zn +100 mg vitamin C +75 mg vitamin E	No significant difference: Total cholesterol, LDL-c, triglycerides Increase: Serum vitamin E, LS-α-TOH, HDL-c, apo A1	3 months
Maktabi et al., 2018 (34)	30.1 ± 5.9 (intervention) 31.5 ± 3.2 (placebo)	F	30/30	Gestational diabetes mellitus	250 mg magnesium oxide daily +400 IU vitamin E daily	No significant difference: HDL-c levels Decrease: FPG, serum insulin levels, HOMA-IR, triglycerides, VLDL-c, total-c, LDL-c Increase: QUICKI	6 weeks
Jamilian et al., 2019 (32)	29.2 ± 7.2 (intervention) 28.3 ± 3.8 (placebo)	F	30/30	Polycystic ovary syndrome	250 mg magnesium as magnesium oxide daily +400 mg vitamin E daily	No significant difference: HDL-c, LDL-c Decrease: serum insulin levels, HOMA-IR, serum triglycerides, VLDL-cholesterol Increase: serum magnesium, QUICKI	12 weeks
Shokrpour et al., 2019 (36)	27.2 ± 7.1 (intervention) 26.0 ± 3.7 (placebo)	F	30/30	Polycystic ovary syndrome	250 mg magnesium as magnesium oxide daily +400 IU vitamin E daily	No significant difference: total testosterone, SHBG, plasma GSH, MDA Decrease: hirsutism, hs-CRP Increase: magnesium, plasma NO, TAC levels	12 weeks
Afzali et al., 2019 (37)	57.2 ± 11.0 (intervention) 55.5 ± 4.9 (placebo)	F/M	29/28	Grade 3 diabetic foot ulcer	250 mg magnesium oxide daily +400 IU vitamin E daily	No significant difference:total cholesterol levels Decrease: triglycerides, LDL-c, hs-CRP, MDA, FPG, insulin, insulin resistance, HbA1c Increase: insulin sensitivity, HDL-c, TAC	12 weeks
Jamilian et al., 2019 (32)	27.7 ± 4.0 (intervention) 29.1 ± 4.1 (placebo)	F	30/30	Gestational diabetes mellitus	100 mg magnesium, 4 mg zinc, 400 mg calcium, 200 IU vitamin D twice a day	Decrease: FPG, hs-CRP, Plasma MDA concentrations Increase: Magnesium, Zinc, Calcium, TAC levels	6 weeks
Kheyruri et al., 2021 (28)	41.00 ± 49.00 (intervention) 42.00 ± 51.00 (placebo)	F	42/41	Middle-aged, Vitamin D deficiency, BMI ≥ 25 kg/m^**2**^	a 250-mg magnesium tablet daily +a 50,000-IU vitamin D soft gel weekly	No significant difference: Magnesium, IL-6, TNF-α Decrease: hs-CRP Increase: 25(OH)D	8 weeks
Abiri et al., 2022 (31)	34.40 ± 9.39 (intervention) 34.36 ± 9.36 (placebo)	F	25/25	Obese, Mild to moderate depressive symptoms	a 250-mg magnesium tablet daily + a 50,000 IU vitamin D soft gel weekly	Decrease: Weight, BMI, WC, BDI-II, TNF-α, IL-6, hs-CRP Increase: 25(OH)D, Magnesium, BDNF, SIRT1	8 weeks
Cheung et al., 2022 (25)	45.3 ± 13.5 (intervention) 41.0 ± 11.2 (placebo)	F/M	21/23	Overweight and obese	360 mg magnesium glycinate daily +1,000 IU vitamin D3 daily	No significant difference: IL-6, CRP Increase: 25(OH)D	12 weeks

F, female; M, male; IU, International Unit; SD, Standard Deviation; 25(OH)D, 25-hydroxyvitamin D; BMI, body mass index; WC, waist circumference; BDI-II, Beck Depression Inventory-II; BDNF, brain-derived neurotrophic factor; SIRT1, Sirtuin1; Mg, magnesium; hs-CRP, hypersensitive-C-reactive protein; CRP, C-reactive protein; TNF-α, tumor necrosis factor alpha; IL, interleukin; FPG, fasting plasma glucose level; MDA, malondialdehyde; TAC, total antioxidant capacity; LDL-c, low density lipoprotein cholesterol; LS-α-TOH, lipid standardized-α-tocopherol; HDL-c, high density lipoprotein cholesterol; Apo A1, apolipoproteins A1; HOMA-IR, homeostatic model of assessment for insulin resistance; VLDL-c, very low density lipoprotein-cholesterol; QUICKI, quantitative insulin sensitivity check index; SHBG, sex hormone-binding globulin; GSH, glutathione; NO, nitric oxide; HbA1c, hemoglobin A1c; m^2^, square metre; kg, kilogram; mg, microgram; ng, nanogram; mL, millilitre.

### 3.3 Risk of bias assessment

**Figure 2:**
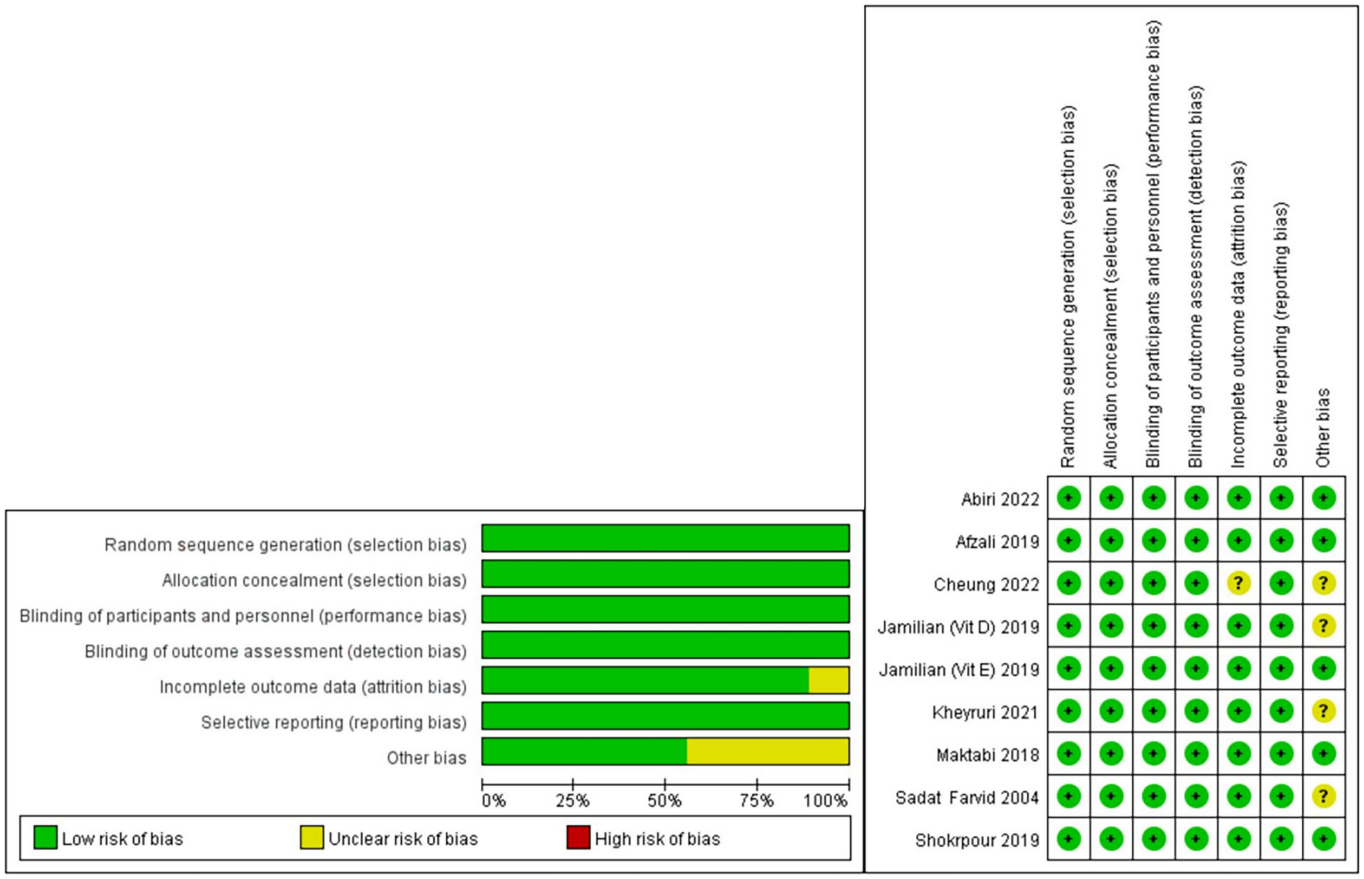
Assessment of the risk of bias for the included studies.

The risk of bias evaluation results are presented in Figure 2. In all studies conducted, a low risk of bias was observed with regard to random sequence generation, allocation concealment, blinding of participants, personnel, and outcome assessment, as well as selective reporting. Considering incomplete outcome data, eight studies demonstrated low risk of bias, while the study by Cheung et al. (25) exhibited an unclear risk of bias. In some cases, discrepancies were noted in the reasons for exclusion and attrition among individuals in the two groups; however, the definitive impact of this inconsistency could not be determined. Upon analyzing other potential biases, four studies (25, 28, 32, 33) had an uncertain risk of bias, and the other five had a low risk of bias.

### 3.4 Meta-analysis results

#### 3.4.1 Effect of co-supplementation on 25(OH)D

**Figure 3:**
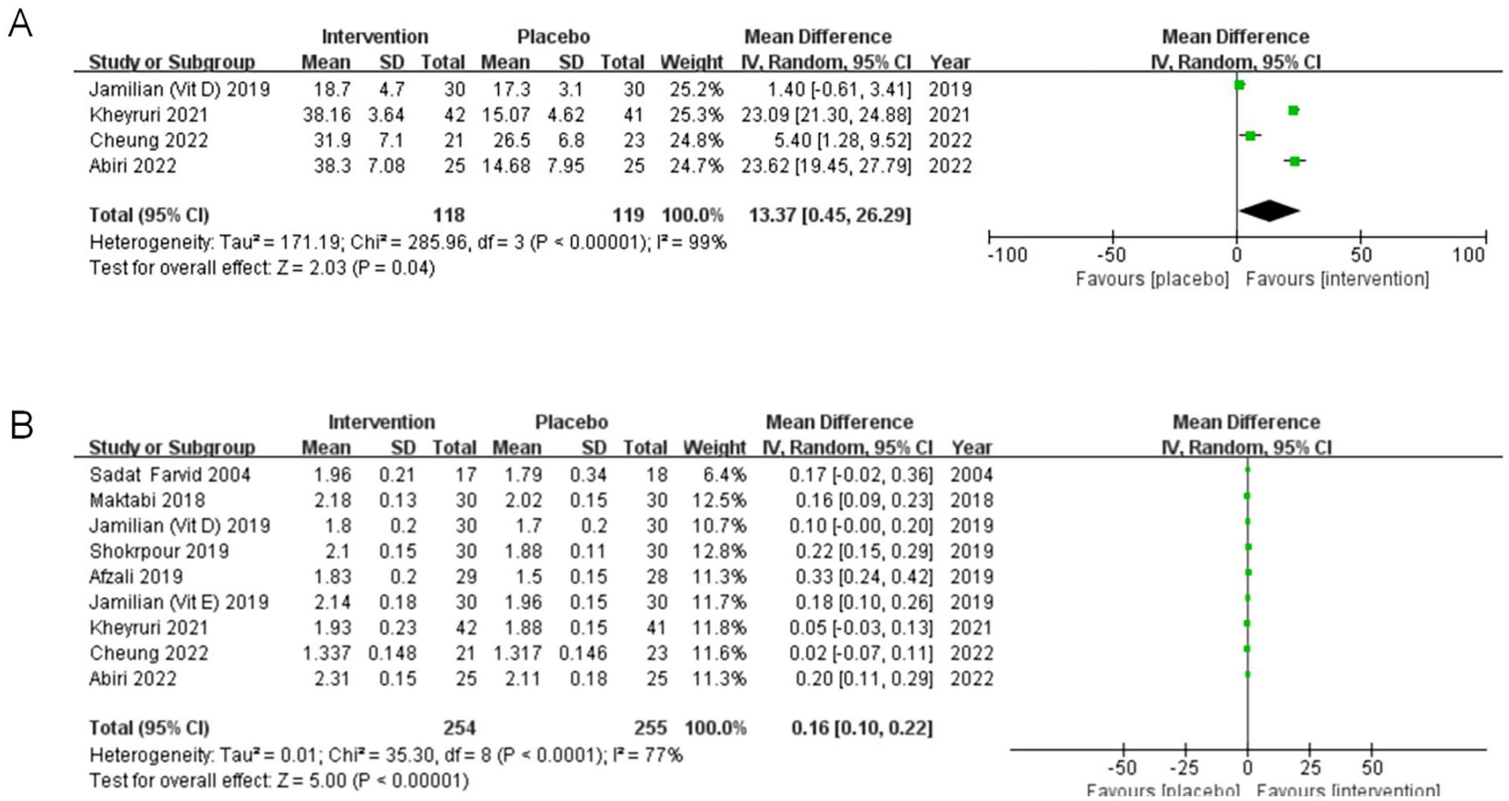
**(A)** Meta-analysis forest plot of the effect of magnesium and vitamin D co-supplementation on serum 25-hydroxyvitamin D levels. **(B)** Meta-analysis forest plot of the effect of magnesium and vitamin D/E co-supplementation on serum magnesium levels.

A total of 237 participants were included from four studies (25, 28, 31, 32) providing data on serum 25(OH)D. Baseline mean and median serum 25(OH)D levels in the studies were all below 30 ng/mL. In comparison with the control group, serum 25(OH)D levels in co-supplementation group increased significantly at the end of the intervention (MD: 13.37, 95% CI: 0.45, 26.29, *p* = 0.04, I^2^ = 99%) (Figure 3A).

#### 3.4.2 Effect of co-supplementation on magnesium

Nine studies (25, 28, 31–37), involving 509 participants with obesity or being overweight, were included into the meta-analysis focusing on serum magnesium. The quality assessment of the included studies was considered satisfactory. The findings demonstrated a notable rise in serum magnesium concentrations post-supplementation (MD: 0.16, 95%CI:0.10, 0.22, *p* < 0.00001, I^2^ = 77%) (Figure 3B).

#### 3.4.3 Effect of co-supplementation on hypersensitive-C reactive protein

**Figure 4:**
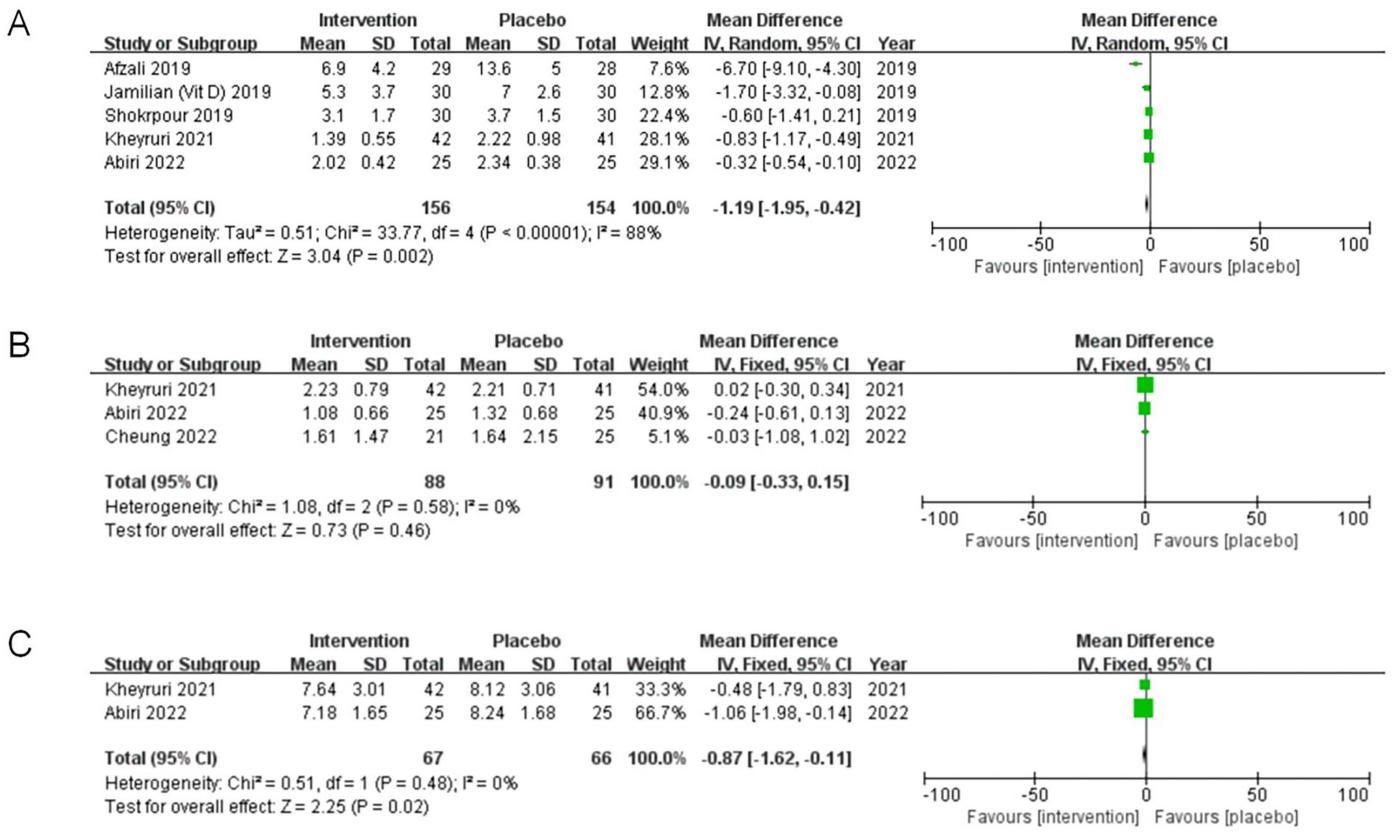
**(A)** Meta-analysis forest plot of the effect of magnesium and vitamin D/E co-supplementation on serum hypersensitive-C reactive protein levels. **(B)** Meta-analysis forest plot of the effect of magnesium and vitamin D co-supplementation on serum interleukin-6 levels. **(C)** Meta-analysis forest plot of the effect of magnesium and vitamin D co-supplementation on serum tumor necrosis factor-α levels.

The combined outcome of five separate studies (28, 31, 32, 36, 37), involving a total of 310 patients, demonstrated a significant impact of co-supplementation for a duration of 6–12 weeks on reducing serum hs-CRP levels (MD: −1.19, 95%CI: −1.95, −0.42, *p* = 0.002, I^2^ = 88%) (Figure 4A).

#### 3.4.4 Effect of co-supplementation on interleukin-6

Three independent studies (25, 28, 31), which focused on 177 patients with inadequate levels of vitamin D, were included to receive magnesium and vitamin D co-supplementation (MD: −0.09, 95%CI: −0.33, 0.15, *p* = 0.46) (Figure 4B). Overall, there were no significant differences among the groups. There was no heterogeneity among these findings (I^2^ = 0%).

#### 3.4.5 Effect of co-supplementation on tumor necrosis factor-*α*

A meta-analysis of two studies (28, 31) investigated the impact of co-supplementation of magnesium and vitamin D on the levels of TNF-α in 133 patients. There was a significant decrease in serum TNF-α levels (MD: −0.87, 95%CI: −1.62, −0.11, *p* = 0.02) (Figure 4C). The analysis did not reveal any heterogeneity.

#### 3.4.6 Effect of co-supplementation on triglyceride levels

**Figure 5:**
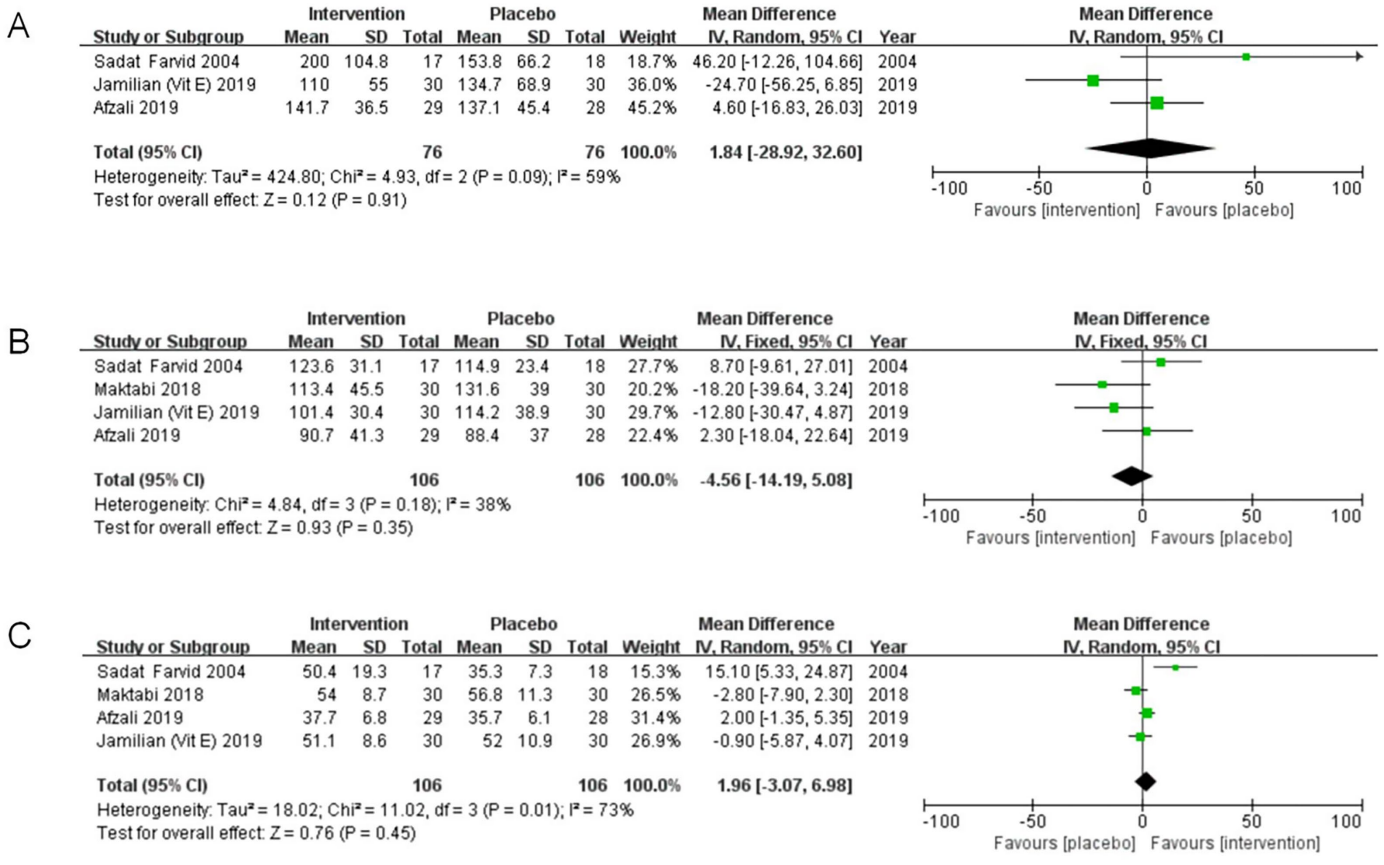
**(A)** Meta-analysis forest plot of the effect of magnesium and vitamin E co-supplementation on serum triglyceride levels. **(B)** Meta-analysis forest plot of the effect of magnesium and vitamin E co-supplementation on serum low density lipoprotein cholesterol levels. **(C)** Meta-analysis forest plot of the effect of magnesium and vitamin E co-supplementation on serum high density lipoprotein cholesterol levels.

A meta-analysis involving three studies (33, 35, 37) investigated the impact of co-supplementation of magnesium and vitamin E on TG levels among 152 participants. Our findings indicated no statistical significance in TG levels (MD = 1.84, 95%CI: −28.92, 32.60, *p* = 0.91, I^2^ = 59%)(Figure 5A).

#### 3.4.7 Effect of co-supplementation on low density lipoprotein cholesterol

The results derived from four distinct studies (33–35, 37), including a total of 212 participants, indicated that co-supplementation of magnesium and vitamin E for 6 to 12 weeks did not significantly impact the decline in serum LDL-c levels (MD: −4.56, 95%CI: −14.19, 5.08, *p* = 0.35, I^2^ = 38%) (Figure 5B).

#### 3.4.8 Effect of co-supplementation on high density lipoprotein cholesterol

In this meta-analysis, data from four independent studies were incorporated (33–35, 37), which collectively examined 212 obese or overweight individuals with co-supplementation of magnesium and vitamin E (MD: 1.96, 95%CI: −3.07, 6.98, *p* = 0.45, I^2^ = 73%) (Figure 5C). Thus, the findings indicated no statistically significant differences among the groups.

### 3.5 Subgroup analysis results

#### 3.5.1 Subgroup analysis of 25(OH)D

The findings from the subgroup analysis indicate that the impact of co-supplementation with magnesium and vitamin D on serum 25(OH)D levels may vary according to dosage.

**Figure 6:**
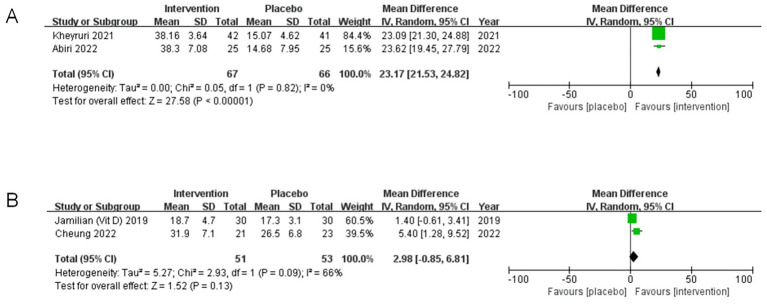
Subgroup analyses of 25-hydroxyvitamin D(25(OH)D). **(A)** Studies with therapeutic dosage vitamin D supplementation. **(B)** Studies with supplementary dosage or less vitamin D supplementation.

For participants receiving therapeutic doses of vitamin D, the analysis demonstrated a significant effect (MD = 23.17, 95% CI: 21.53, 24.82, *p* < 0.00001, I^2^ = 0%) (Figure 6A). Conversely, in participants who received supplementary doses or lower amounts of vitamin D, two studies reported no significant effect (MD = 2.98, 95% CI: −0.85, 6.81, *p* = 0.13, I^2^ = 66%) (Figure 6B).

In summary, the results suggest that administering a therapeutic dose of 25(OH)D substantially increases serum levels of 25(OH)D, while it appears ineffective for those taking supplementary doses or lower quantities. This indicates that the efficacy of co-supplementation regarding 25(OH)D levels may be influenced by the dosage administered.

#### 3.5.2 Subgroup analysis of magnesium

The results of the subgroup analysis indicate that the heterogeneities among subgroups decrease when categorized by the types of vitamin supplements.

**Figure 7:**
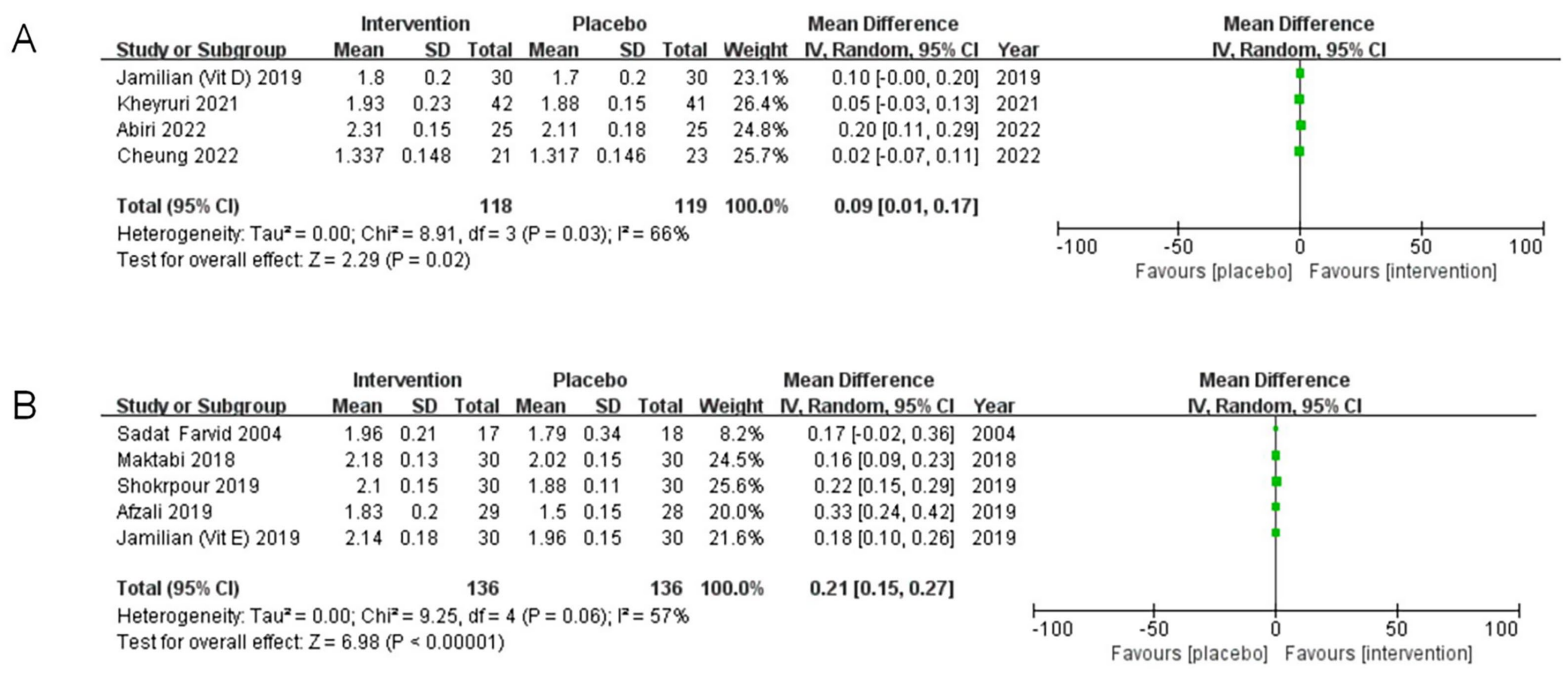
Subgroup analyses of magnesium (Mg). **(A)** Studies with magnesium and vitamin D co-supplementation. **(B)** Studies with magnesium and vitamin E co-supplementation.

In participants who received magnesium in conjunction with vitamin D, four studies reported a significant effect (MD: 0.09, 95% CI: 0.01, 0.17, *p* = 0.02, I^2^ = 66%) (Figure 7A). In contrast, among those taking magnesium with vitamin E, five studies revealed a substantial effect (MD: 0.21, 95% CI: 0.15, 0.27, *p* < 0.00001, I^2^ = 57%) (Figure 7B).

These findings suggest that the specific vitamins combined with magnesium do not change the initial conclusion—whether it is vitamin D or E; however, they may introduce a potential source of variability.

#### 3.5.3 Subgroup analysis of hypersensitive-C reactive protein

The results from the subgroup analysis indicate that the effects of co-supplementation on serum hypersensitive-C reactive protein levels vary among different subgroups.

**Figure 8:**
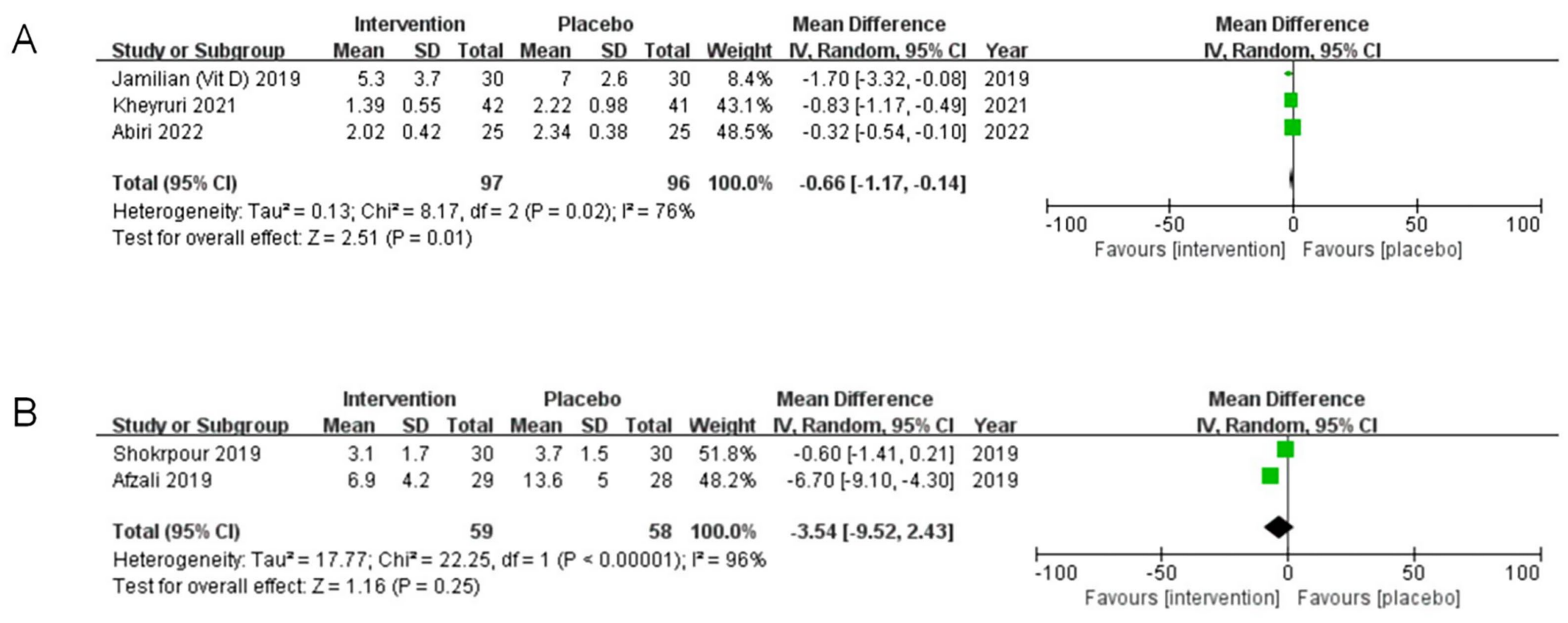
Subgroup analyses of hypersensitive-C reactive protein (hs-CRP). **(A)** Studies with magnesium and vitamin D co-supplementation. **(B)** Studies with magnesium and vitamin E co-supplementation.

The outcomes of the subgroup analysis concerning magnesium co-supplemented with vitamins D/E on serum hs-CRP levels are illustrated in Figures 8A,B. In the study population, a significant reduction in serum hs-CRP levels was observed in participants receiving magnesium and vitamin D compared to those in the placebo group (MD = −0.66, 95% CI: −1.17, −0.14, *p* = 0.01, I^2^ = 76%) (Figure 8A). Conversely, no statistically significant differences were found in subjects receiving magnesium with vitamin E supplementation (MD = −3.54, 95% CI: −9.52, 2.43, *p* = 0.25, I^2^ = 96%) (Figure 8B).

In summary, the results suggest that the effect of co-supplementation with magnesium and vitamins D/E on serum hs-CRP levels may depend on the specific type of vitamin, indicating that different vitamins may influence serum hs-CRP to varying extents.

### 3.6 Sensitivity analysis

Sensitivity analyses were conducted on the study results to evaluate their stability. A stepwise elimination approach was employed for outcome measures that exhibited significant heterogeneity, assessing whether the combined effect size estimates from the remaining studies still fell within the 95% confidence interval of the overall effect size. The results indicated that the removal of any individual study did not substantially alter the overall conclusions, thereby affirming the validity of the research findings (Supplementary Tables 2A–E). Regarding the impact of co-supplementation on serum magnesium levels, a sensitivity analysis was performed by systematically excluding specific studies. This analysis revealed that the research conducted by Afzali (37) was the principal contributor to the observed heterogeneity. Its exclusion reduced heterogeneity to I^2^ = 66%, while the effect size remained relatively constant (MD: 0.14, 95% CI: 0.08, 0.19, *p* < 0.00001). Similarly, when evaluating the effects of co-supplementation on serum levels of hypersensitive C-reactive protein, the same methodology indicated that Afzali’s study (37) was again the leading source of heterogeneity. The removal of this study resulted in a decrease in heterogeneity to I^2^ = 64%, while the effect size remained largely stable (MD: −0.63, 95% CI: −1.04, −0.22, *p* = 0.003). In assessing the effect of co-supplementation on serum triglyceride levels, omitting Jamilian’s study (35) led to a reduction of heterogeneity to 42%, with the effect size remaining stable (MD: −16.15, 95% CI: −20.36, 52.66, *p* = 0.39). Furthermore, in examining the influence of co-supplementation on serum high-density lipoprotein cholesterol levels, the research by Farvid (33) was identified as the primary source of heterogeneity. Its exclusion resulted in a reduction in heterogeneity to 24%, while the effect size continued to exhibit significant stability (MD: −0.01, 95% CI: −2.88, 2.86, *p* = 1.00). Nevertheless, in terms of the impact of co-supplementation on serum 25(OH)D levels, changing the inclusion of any studies did not have a notable effect on heterogeneity, which continued to be high (97, 98, 99, 99%).

### 3.7 Publication bias

**Figure 9:**
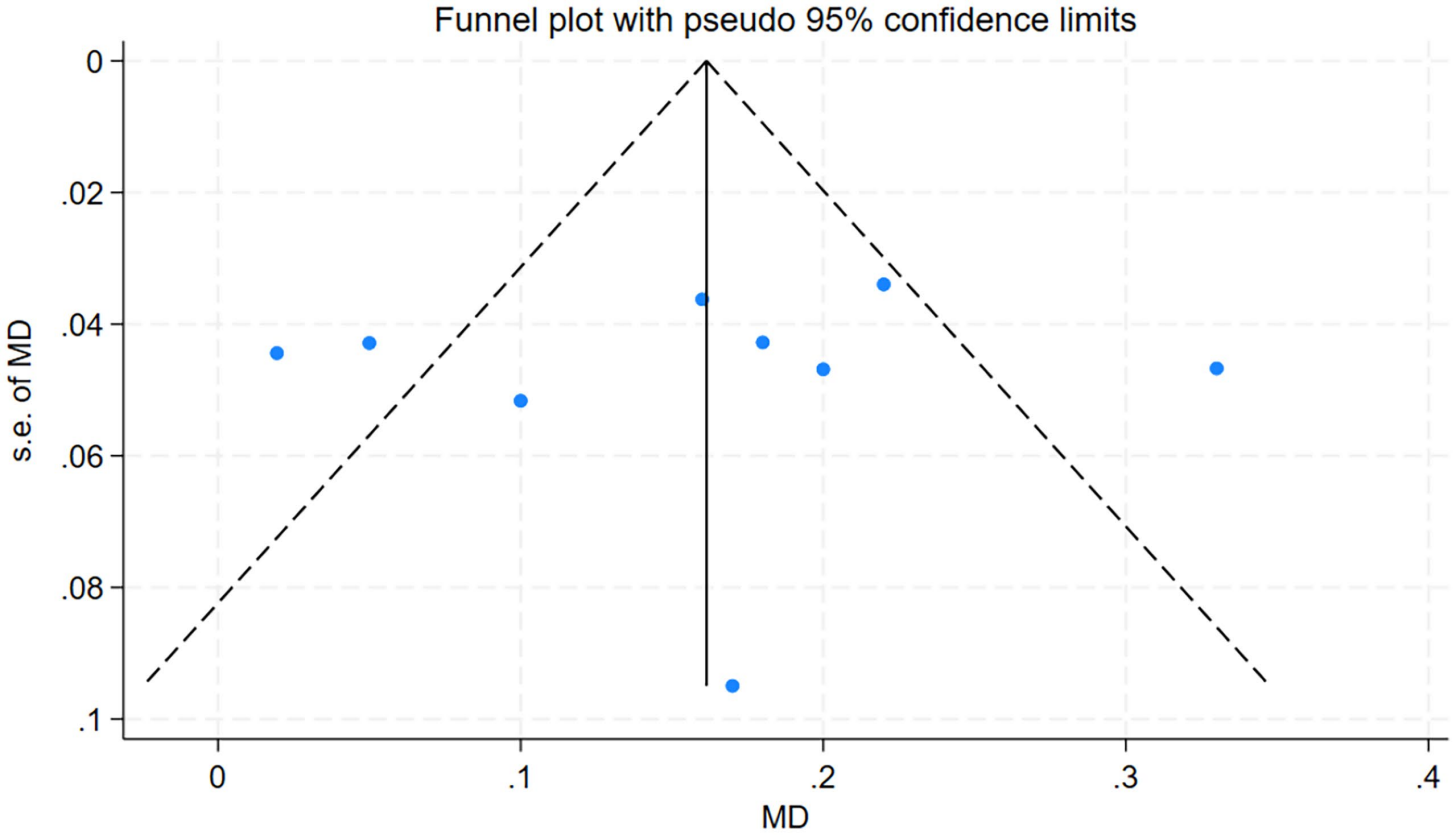
Funnel plot.

The funnel plot illustrates the assessment results regarding the risk derived from nine studies that investigated the effects of co-supplementing magnesium with vitamins D/E on serum magnesium concentrations. The findings indicate that significant publication bias is not present in these nine studies (Figure 9). Furthermore, the application of Egger’s test revealed no evidence of publication bias across all measures in this analysis (Supplementary Table 3).

## 4 Discussion

This study marks the first comprehensive review and meta-analysis of the inflammatory effects of co-supplementation of vitamin D/E and magnesium based on evidence from RCTs. Findings from the analysis reveal that co-supplementation of these two essential nutrients leads to increased levels of 25-(OH)D and serum magnesium in the body. Additionally, it results in a significant reduction in serum levels of hs-CRP and TNF-*α*, showcasing the potential benefits of this combined supplementation. Notably, the study did not demonstrate a statistically significant effect of co-supplementation of vitamin D/E and magnesium on IL-6 levels. Further research is warranted to explore the mechanisms behind these effects and to understand the implications for inflammation-related health outcomes.

Traditionally, the benefits of vitamin D supplementation include preventing and treating sarcopenia and osteoporosis (38). Muscle deterioration and osteoporosis are associated with vitamin D deficiency, increasing the risk of fractures. Studies also indicate that a lack of vitamin D can lead to a decrease in skeletal muscle mass and strength (39). Conflicting research results are attributed to variations in individuals, vitamin D doses, and environmental factors (40). The recommended daily doses of vitamin D range from 200 to 2000 IU, with a daily intake of 1,000 IU suggested for those at high risk of deficiency (41). However, excessive vitamin D supplementation can result in symptoms like nausea, vomiting, weakness, and potential kidney damage. Current research shows a positive impact of vitamin D supplementation on specific musculoskeletal functions (42). However, the effectiveness of vitamin D supplementation is often not satisfactory; this can be attributed to factors such as patient compliance or variations in intestinal absorption problems (43). To achieve the desired effect, it is often necessary to supplement vitamin D along with other trace elements. Palacios et al. (42) carried out a research study on vitamin D supplementation during pregnancy to determine if administering vitamin D alone or with calcium or other vitamins and minerals to pregnant women can effectively enhance maternal and neonatal health. The addition of vitamin D to the diet of pregnant women could potentially reduce the risk of pre-eclampsia, gestational diabetes, and low birthweight, as well as lower the incidence of severe postpartum hemorrhage. Nevertheless, there may be minimal to no impact on the risk of premature delivery before the completion of 37 weeks of gestation period. On the other hand, the combined administration of vitamin D and calcium to expectant mothers has shown promise in lowering the chances of developing pre-eclampsia (42). In the research conducted by Bouderlique et al., the effects of extended use of vitamin D supplements, in conjunction with or without a calcium-enriched meal plan, on the formation of Randall’s plaque in a rodent model Abcc6 mice were examined. This distinctive experiment shed light on the possible combined hazard linked to taking vitamin D supplements and consuming calcium in the progression of Randall’s plaque (44). In their review, Grant et al. explored the impact of vitamin D on respiratory tract infections, specifically influenza and coronavirus disease-2019. The potential benefits of vitamin D supplementation in reducing the risk of infections were also examined. Findings from observational studies and clinical trials on the effectiveness of vitamin D supplementation in decreasing influenza risk were reported to be inconsistent (45). Another review and analysis of aggregate data from RCTs were undertaken to assess the impact of vitamin D supplementation on preventing acute respiratory infections and reported that compared to placebo, vitamin D supplementation decreased the overall risk of acute respiratory infections (46).

Vitamin E has a significant role as an antioxidant, which may help prevent cardiovascular disease (CVD) (47). Epidemiological studies have demonstrated that increased vitamin E intake may lead to a reduced risk of developing CVD. However, this beneficial effect is only shown among individuals who used high doses of vitamin E supplements for over 2 years (48) Vitamin E can also enhance T-cell-mediated functions in the aging population, and contributes to both innate and adaptive immune responses. Research reports that supplementing 800 IU of vitamin E per day for 1 month in healthy older individuals resulted in notable improvements in both *in vivo* and ex vivo indices of T cell functions (≥60 years old) (49). Furthermore, vitamin E also suppress inflammatory factors that indirectly help in the regulation of T cells (11). Vitamin E supplementation in older adults has been associated with a reduction in levels of various inflammatory markers, including TNF-*α* and IL-6, particularly in response to pathogens (50, 51). Additionally, vitamin E activates the AMP-activated protein kinase (AMPK) pathway, leading to the phosphorylation of acetyl-CoA carboxylase; it helps to diminish fatty acid synthesis. Bai et al. (52) demonstrated that aerobic exercise and vitamin E supplementation in rat subjects significantly improved conditions resembling non-alcoholic fatty liver disease.

Magnesium facilitates various crucial functions in the body, particularly in relation to muscle mass and strength, including the production of energy, transportation of substances across cell membranes, maintenance of electrolyte balance, and muscle contractions and relaxations (53). One randomized controlled trial showed improved physical performance of healthy women (average age 71.5 ± 5.2 years) when they combined magnesium supplementation (300 mg/day) with a light exercise program (54). However, another separate RCT found that taking 250 mg of magnesium daily for 8 weeks did not increase handgrip strength and knee extension strength relative to a placebo group (53). However, when middle-aged women with low vitamin D levels took a combination of vitamin D (50,000 IU per week) and magnesium (250 mg daily) supplements for 8 weeks, their muscle strength, function, and certain inflammatory markers were positively affected (28). Along with its role in facilitating muscle relaxation, magnesium is of utmost importance in the synthesis of proteins and ATP. Furthermore, it aids in the transfer of calcium and potassium ions through cell membranes, a vital process for neuronal activity as well as muscle contraction (55). The responses to magnesium supplementation could be due to the bioavailability of magnesium from daily intake of magnesium oxide (56). The response to supplementation might be determined by the elemental magnesium content as well as the bioavailability of the magnesium supplement. Even though magnesium oxide has a higher elemental magnesium content compared to other supplement forms, it possesses lower bioavailability (57). Additionally, the response to magnesium supplementation could be impacted by the baseline magnesium level or baseline muscle strength. People with lower baseline serum magnesium levels or baseline muscle strength usually exhibit a more favorable response to supplementation of magnesium (28). Concurrently, researchers discovered that patients experiencing a lack of vitamin D demonstrated a notable rise in serum 25(OH)D (13.0 ± 5.06 ng/mL) when provided with co-supplements of both vitamin D and magnesium. This increase was markedly greater compared to the group receiving only vitamin D supplementation, which resulted in a serum 25(OH)D level of (2.39 ± 0.46 ng/mL) (25). Studies (21) have suggested that magnesium plays a critical function in the creation and breakdown of parathyroid hormone and vitamin D. Specifically, magnesium influences the actions of three vital enzymes that directly impact the levels of 25(OH)D (58) These enzymes include 25-hydroxylase, 1α-hydroxylase, and 24-hydroxylase. Magnesium also influences the operation of vitamin D-binding protein (VDBP). All these aforementioned enzymes and VDBP necessitate magnesium for their optimal operation. According to the literature and the outcomes of our meta-analysis, it is recommended to supplement vitamin D and magnesium jointly for individuals experiencing vitamin D deficiency. This supplementation can increase the concentration of serum magnesium ions in patients and potentially enhance the serum 25-(OH)D levels.

The effect of co-supplementation of vitamin D and magnesium in reducing serum hs-CRP levels was large and significant in our meta-analysis. Hs-CRP, as an effective means of identifying potential systemic inflammation, proves to be valuable. Irrespective of conventional risk factors, increased hs-CRP levels in individuals without apparent symptoms can function as a significant indicator of potential heart attack, stroke, diabetes, and cardiovascular fatality. By potentially assisting in therapy selection and foreseeing vascular incidents, hs-CRP levels hold potential significance (59) Kim et al. investigated the correlation between serum magnesium and various inflammatory markers such as hs-CRP, IL-6, and fibrinogen (60) The study sample comprised 4,497 individuals aged 18–30 years, from the United States. The outcomes demonstrated an inverse relationship between serum magnesium concentrations and hs-CRP levels. This investigation delved into the possible association between serum magnesium and inflammatory indicators in a young American cohort. This suggests that higher serum magnesium concentrations may be associated with lower hs-CRP levels, indicating a potential anti-inflammatory effect of magnesium (60). There is a clear correlation between inadequate levels of vitamin D and inflammation in overweight persons (61, 62). Various clinical trials have thoroughly assessed the impact of vitamin D on managing inflammation. An analysis of 40 female locals from the neighborhood discovered that a solitary dose of vitamin D (200,000 IU) noticeably lowered hs-CRP levels following a 4-week timeframe. Particularly, administering a high dose of vitamin D3 lowered inflammatory indicators and heightened the overall antioxidant capability in older females facing vitamin D insufficiency (63).

The interplay between vitamin D and magnesium has garnered significant attention due to their potential synergistic effects on vascular endothelial function, a critical aspect of cardiovascular health. Recent research highlights the importance of dietary magnesium intake in modulating the relationship between vitamin D levels and various cardiovascular parameters, including blood pressure. A study utilizing data from the National Health and Nutrition Examination Survey (NHANES) demonstrated that higher magnesium intake significantly modified the negative relationship between vitamin D and systolic blood pressure (SBP), suggesting that magnesium may amplify the beneficial effects of vitamin D on vascular health (64). Furthermore, the interaction between these two nutrients appears to extend to metabolic health, with evidence suggesting that magnesium intake can influence the association between vitamin D levels and insulin resistance, thereby impacting overall cardiovascular risk profiles (65). In addition to their direct effects on endothelial cells, both magnesium and vitamin D are implicated in the modulation of inflammatory pathways that can adversely affect vascular health. Magnesium deficiency is associated with increased oxidative stress and inflammation, both of which contribute to endothelial dysfunction and cardiovascular disease (66). Conversely, vitamin D has been recognized for its anti-inflammatory properties, which may help mitigate the inflammatory responses that compromise endothelial integrity (67).

In this meta-analysis, we observed that the combined use of vitamin D and magnesium did not significantly impact IL-6 serum levels. Although there is substantial molecular evidence indicating the key role of vitamin D in changing inflammatory markers, the results in adult observational studies are conflicting, and clinical trials mainly target specific disease categories. As a result, the clinical evidence associating vitamin D with inflammation in older individuals is remarkably inconsistent (68). A clinical study conducted by Moslehi et al. investigated the effects of magnesium supplementation on hs-CRP, plasma fibrinogen, and IL-6 levels in healthy middle-aged overweight women. The participants were given 250 mg of magnesium oxide daily for 8 weeks (69). The results of the study showed that compared with baseline values, inflammatory markers such as hs-CRP and IL-6 did not decrease in the magnesium group (63). Although the exact mechanism by which magnesium exerts its anti-inflammatory properties remains largely unraveled, studies suggest that insufficient magnesium may lead to an increase in intracellular calcium, leading to inflammation (70). Among the three RCTs included in this meta-analysis to enroll and analyze data on the indicator of IL-6, only one RCT reported a significant reduction in IL-6 levels after vitamin D and magnesium supplementation. The remaining RCTs did not report any significant differences. Further clinical studies in the future should examine changes in inflammatory factors, especially IL-6, following simultaneous intake of vitamin D and magnesium.

Although only two RCTs provided clinical data in this meta-analysis, combined vitamin D and magnesium supplementation had a significant effect on reducing serum TNF-*α* levels. TNF-α is a pro-inflammatory cytokine, known for its significant involvement in inflammation, apoptosis, and cancer. It plays a crucial role in regulating vasodilatation, formation of edema, leukocyte adhesion, blood coagulation, and oxidative stress (71). TNF-*α* is closely associated with the development of chronic inflammation and cancer. Inflammaging, which is chronic, low-grade inflammation that occurs with advanced age, is linked to increased levels of TNF-α (72). King and colleagues found a noteworthy association between decreased magnesium levels in the bloodstream and elevated levels of TNF-α (73). Additionally, studies have indicated that 25-(OH)D and 1,25-(OH)2D3 are capable of suppressing human monocytes-derived TNF-α production in macrophages (22). Vitamin D has also been demonstrated to enhance endothelial barrier integrity and counteract TNF-α-induced inflammatory conditions (74). Furthermore, vitamin D treatment has also been observed to reverse oxidative stress, improve antioxidant status, and alleviate the inflammatory response induced by TNF-α (75). We anticipate that further clinical experimental studies in the future will provide evidence supporting the claim that vitamin D and magnesium co-supplementation can effectively decrease the levels of serum TNF-α and other inflammatory factors in patients.

In our meta-analysis subgroup analysis, magnesium and vitamin E co-supplementation did not result in a reduction of inflammatory factors in obese patients. However, there is a substantial body of literature supporting the existence of this phenomenon. Notably, a study of effects of magnesium and vitamin E co-supplementation on hormonal status and biomarkers of inflammation and oxidative stress in women with polycystic ovary syndrome demonstrated that co-supplementation for 12 weeks significantly decreased serum hs-CRP levels (36). When examining magnesium and vitamin E individually, one study indicated that magnesium supplementation as magnesium oxide at a dosage of 250 mg/day, administered over 8 weeks, did not affect inflammatory markers in middle-aged overweight women. In contrast, vitamin E demonstrates a more pronounced anti-inflammatory effect; a meta-analysis by Saboori et al. found that vitamin E supplementation significantly reduced CRP levels (76). The anti-inflammatory effects of magnesium supplements may be due to the antagonistic action of magnesium against calcium and their ability to inhibit nuclear factor-kappa B (NF-κB) (77).

Numerous studies have investigated the joint supplementation of magnesium and vitamin E, and found positive results in reducing indicators of lipid metabolism in patients. However, our meta-analysis found no significant reductions of TG, LDL-c, and HDL-c levels in obese patients. In an animal study, vitamin E supplementation over a period of 20 weeks effectively reduced serum levels of TG and VLDL-cholesterol, but did not affect the profiles of other lipids (78). Additionally, magnesium intake may lower circulating levels of TG and VLDL-cholesterol by enhancing the excretion of fecal fat and increasing the activity of lipoprotein lipase (79). Another clinical study indicated that magnesium supplementation over a duration of 4 months led to a reduction in TG levels among pre-diabetic patients with hypomagnesemia (80). Additionally, Ekhlasi et al. (81) reported that symbiotic and vitamin E co-supplementation resulted in decreased levels of TG, total cholesterol, and LDL-c, while having no significant impact on HDL-c concentrations. Consistent with our findings, a meta-analysis conducted by Xu et al. (82) reported no significant effects of vitamin E supplementation on lipid profiles. Concurrently, magnesium supplementation failed to improve lipid levels in both diabetic and non-diabetic individuals (83).

Although magnesium or vitamin E supplementation alone have a positive regulatory effect on lipid metabolism, the effectiveness of their combined use may be influenced by multiple factors. Research has shown that supplementing high doses of magnesium (such as the medium to high dose group) or vitamin E (such as 200–400 mg/kg) alone can significantly reduce cholesterol (TC), low density lipoprotein cholesterol(LDL-c), and triglycerides. However, when combined supplementation, if the dosage is not optimized, it may not be possible to produce additive or synergistic effects. High doses of magnesium may interfere with the absorption of fat soluble vitamins such as vitamin E (84). Another possible reason could be the redundancy of oxidative stress regulation. Vitamin E reduces lipid synthesis and enhances antioxidant capacity by activating the Nrf2/CES1 pathway, while magnesium affects lipid metabolism by regulating enzyme activity. The interaction between inflammation and lipid metabolism should also be considered. Vitamin E reduces inflammation by inhibiting the NF-κB pathway, while magnesium may affect the inflammatory response through other pathways, such as regulating cytokines. If the inflammatory state is not adequately regulated, combined supplementation may not further improve lipid metabolism (85, 86).

It is inevitable that some specific diseases such as polycystic ovary syndrome (PCOS) and gestational diabetes mellitus limit the inclusion of male participants. In sensitivity analysis, we found that gender is a possible source of heterogeneity. In the study of triglyceride, after excluding Jamilian’s study, the heterogeneity of the study decreased from 59 to 42%. Moreover, in the study of hs-CRP, IL-6, TG, HDL-c, the conclusion did not change either in F/M or F group, suggesting that the combined supplementation of Mg and vitamin E may not significantly improve lipid metabolism in terms of mechanism. On the other hand, magnesium and vitamin D co-supplementation may also improve inflammatory status for populations of different genders. However, we found that the combined supplementation of magnesium and vitamin E had a more significant effect on reducing low-density lipoprotein cholesterol (LDL-C) in women in the summary analysis of Jamilian’s (35) and Maktabi’s (34) studies. We thought that it mainly related to some synergistic mechanisms. The first reason can be the improvement of insulin resistance. In women with PCOS and gestational diabetes, magnesium combined with vitamin E significantly reduced fasting insulin level (−2.93μIU/mL) and HOMA-IR index (−0.78), while improving insulin sensitivity index (QUICKI+0.01) (34, 35). The improvement of insulin resistance can reduce the secretion of very low-density lipoprotein (VLDL) in the liver, thereby lowering the level of LDL-C precursor (34, 35, 87). The above is based on a small sample size, more researches are required to solve the problem of gender imbalance and generate more convincing conclusions.

Due to strict inclusion criteria, this meta-analysis included only nine RCTs with a small overall patient population. Future studies will include more patient data to enhance the statistical significance of the meta-analysis. Gender imbalance was noted in the RCTs included in this study, with three studies with no gender restrictions and six articles exclusively featuring female patients. Furthermore, subgroup analyses were conducted separately for participants receiving magnesium and vitamin D, as well as magnesium and vitamin E to evaluate the impact of magnesium and vitamin D/E supplementation on inflammatory marker hs-CRP. In addition, the variations in patient demographics, such as obesity, gestational diabetes, type 2 diabetes, polycystic ovary syndrome, and vitamin D deficiency among the included studies induced potential biases. Efforts need be made to include additional literature and mitigate these biases.

## 5 Conclusion

This systematic review and meta-analysis indicates that co-supplementation of vitamin D/E and magnesium in patients may potentially increase serum levels of 25(OH)D and decrease the levels of inflammatory markers like hs-CRP and TNF-*α*. While, the combined supplementation of vitamin E and magnesium ions did not improve lipid metabolism. The heterogeneity across studies remains a critical concern. Variability in study designs, populations, dosing regimens, and duration of interventions contributes to inconsistent outcomes, complicating our ability to draw definitive conclusions about the efficacy and optimal use of these supplements. Future research should therefore focus on specific lipid fractions, oxidative stress markers with longer follow-up and larger scale RCTs, exploring precision nutrition intervention strategies.

## Data Availability

The original contributions presented in the study are included in the article/Supplementary material, further inquiries can be directed to the corresponding author.

## References

[1] Shilin J Hui Y Fang H Wenguo F. Systemic inflammation, neuroinflammation and perioperative neurocognitive disorders. Inflamm Res. (2023) 72:1895–907. doi: 10.1007/s00011-023-01792-2, PMID: 37688642

[2] Volodymyr P Flore S Howard R Charna D. Lipid metabolism around the body clocks. Prog Lipid Res. (2023) 91:101235. doi: 10.1016/j.plipres.2023.101235, PMID: 37187314

[3] González-Muniesa P Mártinez-González MA Hu FB Després JP Matsuzawa Y Loos RJF . Obesity. Nat Rev Dis Primers. (2017) 3:17034. doi: 10.1038/nrdp.2017.34, PMID: 28617414

[4] Martineau AR Thummel KE Wang Z Jolliffe DA Boucher BJ Griffin SJ . Differential effects of oral boluses of Vitamin D2 vs Vitamin D3 on Vitamin D metabolism: a randomized controlled trial. J Clin Endocrinol Metab. (2019) 104:5831–5839. doi: 10.1210/jc.2019-00207 31199458 PMC6797055

[5] Calton EK Keane KN Newsholme P Soares MJ. The impact of vitamin D levels on inflammatory status: a systematic review of immune cell studies. PLoS One. (2015) 10:e0141770. doi: 10.1371/journal.pone.0141770, PMID: 26528817 PMC4631349

[6] John JC William BG Michael FH. Vitamin D and inflammation. Dermatoendocrinol. (2014) 6:e983401. doi: 10.4161/19381980.2014.983401, PMID: 26413186 PMC4580066

[7] R G C M A S M S C. Vitamin D deficiency and chronic lung disease. Can Respir J. (2009) 16:75–80. doi: 10.1155/2009/829130, PMID: 19557213 PMC2706673

[8] Arancibia-Hernández YL Aranda-Rivera AK Cruz-Gregorio A Pedraza-Chaverri J. Antioxidant/anti-inflammatory effect of mg (2+) in coronavirus disease 2019 (COVID-19). Rev Med Virol. (2022) 32:e2348. doi: 10.1002/rmv.2348, PMID: 35357063 PMC9111052

[9] Li R Li Z Huang Y Hu K Ma B Yang Y. The effect of magnesium alone or its combination with other supplements on the markers of inflammation, OS and metabolism in women with polycystic ovarian syndrome (PCOS): a systematic review. Front Endocrinol (Lausanne). (2022) 13:974042. doi: 10.3389/fendo.2022.974042, PMID: 35992132 PMC9389579

[10] Patrícia M Gonçalo Á Ana Carina F Ivo L Aníbal F. Hypomagnesemia: a potential underlooked cause of persistent vitamin D deficiency in chronic kidney disease. Clin Kidney J. (2023) 16:1776–85. doi: 10.1093/ckj/sfad123, PMID: 37915933 PMC10616498

[11] Erin Diane L Simin Nikbin M Dayong W. Regulatory role of vitamin E in the immune system and inflammation. IUBMB Life. (2018) 71:487–94. doi: 10.1002/iub.1976, PMID: 30501009 PMC7011499

[12] Gregor Carpentero B Kiyotaka N Fumiko K Teruo M. Tocotrienol attenuates triglyceride accumulation in Hep G2 cells and F344 rats. Lipids. (2012) 47:471–81. doi: 10.1007/s11745-012-3659-0, PMID: 22367056

[13] Akira S Yuki K Toshiyuki K Teruo M Kiyotaka N. Α-tocopherol attenuates the triglyceride-and cholesterol-lowering effects of rice bran tocotrienol in rats fed a western diet. J Agric Food Chem. (2016) 64:5361–6. doi: 10.1021/acs.jafc.6b02228, PMID: 27295311

[14] Weng-Yew W Leigh CW Chee Wai F Wei Ney Y Lindsay B. Anti-inflammatory γ- and δ-tocotrienols improve cardiovascular, liver and metabolic function in diet-induced obese rats. Eur J Nutr. (2015) 56:133–50. doi: 10.1007/s00394-015-1064-1, PMID: 26446095

[15] Waniek S di Giuseppe R Plachta-Danielzik S Ratjen I Jacobs G Koch M . Association of vitamin E levels with metabolic syndrome, and MRI-derived body fat volumes and liver fat content. Nutrients. (2017) 9:1143. doi: 10.3390/nu9101143, PMID: 29057829 PMC5691759

[16] Arman A Nahid R Reza A Fatemeh S. The role of magnesium in sleep health: a systematic review of available literature. Biol Trace Elem Res. (2022) 201:121–8. doi: 10.1007/s12011-022-03162-1, PMID: 35184264

[17] Sugimoto J Romani AM Valentin-Torres AM Luciano AA Ramirez Kitchen CM Funderburg N . Magnesium decreases inflammatory cytokine production: a novel innate immunomodulatory mechanism. J Immunol. (2012) 188:6338–46. doi: 10.4049/jimmunol.1101765, PMID: 22611240 PMC3884513

[18] Bussière FI Gueux E Rock E Girardeau JP Tridon A Mazur A . Increased phagocytosis and production of reactive oxygen species by neutrophils during magnesium deficiency in rats and inhibition by high magnesium concentration. Br J Nutr. (2002) 87:107–13. doi: 10.1079/bjn2001498, PMID: 11895162

[19] Luis J M M G. Magnesium in critical illness: metabolism, assessment, and treatment. Intensive Care Med. (2002) 28:667–79. doi: 10.1007/s00134-002-1281-y, PMID: 12107669

[20] Inoue I. Lipid metabolism and magnesium. Clin Calcium. (2005) 15:65–76. 16272615

[21] Dai Q Zhu X Manson JE Song Y Li X Franke AA . Magnesium status and supplementation influence vitamin D status and metabolism: results from a randomized trial. Am J Clin Nutr. (2018) 108:1249–58. doi: 10.1093/ajcn/nqy274, PMID: 30541089 PMC6693398

[22] R A R L H L M HJ. 25(OH)D3 and 1.25(OH)2D3 inhibits TNF-α expression in human monocyte derived macrophages. PLoS One. (2019) 14:e0215383. doi: 10.1371/journal.pone.0215383, PMID: 30978243 PMC6461260

[23] Valko M Rhodes CJ Moncol J Izakovic M Mazur M. Free radicals, metals and antioxidants in oxidative stress-induced cancer. Chem Biol Interact. (2006) 160:1–40. doi: 10.1016/j.cbi.2005.12.009 16430879

[24] Andrea R Qi D Sue AS. Essential nutrient interactions: does low or suboptimal magnesium status interact with vitamin D and/or calcium status? Adv Nutr. (2016) 7:25–43. doi: 10.3945/an.115.008631, PMID: 26773013 PMC4717874

[25] Cheung MM Dall RD Shewokis PA Altasan A Volpe SL Amori R . The effect of combined magnesium and vitamin D supplementation on vitamin D status, systemic inflammation, and blood pressure: a randomized double-blinded controlled trial. Nutrition. (2022) 99–100:111674. doi: 10.1016/j.nut.2022.111674, PMID: 35576873

[26] Moher D Liberati A Tetzlaff J Altman DG. Preferred reporting items for systematic reviews and meta-analyses: the PRISMA statement. Open Med. (2009) 3:e123–30. PMC3090117 21603045

[27] Higgins JP Altman DG Gøtzsche PC Jüni P Moher D Oxman AD . The Cochrane collaboration’s tool for assessing risk of bias in randomised trials. BMJ. (2011) 343:d5928. doi: 10.1136/bmj.d5928, PMID: 22008217 PMC3196245

[28] Kheyruri F Sarrafzadeh J Hosseini AF Abiri B Vafa M. Randomized study of the effects of vitamin D and magnesium co-supplementation on muscle strength and function, body composition, and inflammation in vitamin D-deficient middle-aged women. Biol Trace Elem Res. (2020) 199:2523–34. doi: 10.1007/s12011-020-02387-2, PMID: 32955720

[29] Luo D Wan X Liu J Tong T. Optimally estimating the sample mean from the sample size, median, mid-range, and/or mid-quartile range. Stat Methods Med Res. (2016) 27:1785–805. doi: 10.1177/0962280216669183, PMID: 27683581

[30] Wan X Wang W Liu J Tong T. Estimating the sample mean and standard deviation from the sample size, median, range and/or interquartile range. BMC Med Res Methodol. (2014) 14:135. doi: 10.1186/1471-2288-14-135, PMID: 25524443 PMC4383202

[31] Abiri B Sarbakhsh P Vafa M. Randomized study of the effects of vitamin D and/or magnesium supplementation on mood, serum levels of BDNF, inflammation, and SIRT1 in obese women with mild to moderate depressive symptoms. Nutr Neurosci. (2022) 25:2123–35. doi: 10.1080/1028415x.2021.1945859, PMID: 34210242

[32] Jamilian M Mirhosseini N Eslahi M Bahmani F Shokrpour M Chamani M . The effects of magnesium-zinc-calcium-vitamin D co-supplementation on biomarkers of inflammation, oxidative stress and pregnancy outcomes in gestational diabetes. BMC Pregnancy Childbirth. (2019) 19:107. doi: 10.1186/s12884-019-2258-y, PMID: 30922259 PMC6440090

[33] Farvid M Siassi F Jalali M Hosseini M Saadat N. The impact of vitamin and/or mineral supplementation on lipid profiles in type 2 diabetes. Diabetes Res Clin Pract. (2004) 65:21–8. doi: 10.1016/j.diabres.2003.11.009, PMID: 15163474

[34] Maktabi M Jamilian M Amirani E Chamani M Asemi Z. The effects of magnesium and vitamin E co-supplementation on parameters of glucose homeostasis and lipid profiles in patients with gestational diabetes. Lipids Health Dis. (2018) 17:163. doi: 10.1186/s12944-018-0814-5, PMID: 30025522 PMC6053775

[35] Jamilian M Sabzevar NK Asemi Z. The effect of magnesium and vitamin E co-supplementation on glycemic control and markers of cardio-metabolic risk in women with polycystic ovary syndrome: a randomized, double-blind, placebo-controlled trial. Horm Metab Res. (2018) 51:100–5. doi: 10.1055/a-0749-6431, PMID: 30286483

[36] Shokrpour M Asemi Z. The effects of magnesium and vitamin E co-supplementation on hormonal status and biomarkers of inflammation and oxidative stress in women with polycystic ovary syndrome. Biol Trace Elem Res. (2018) 191:54–60. doi: 10.1007/s12011-018-1602-9, PMID: 30565017

[37] Afzali H Jafari Kashi AH Momen-Heravi M Razzaghi R Amirani E Bahmani F . The effects of magnesium and vitamin E co-supplementation on wound healing and metabolic status in patients with diabetic foot ulcer: a randomized, double-blind, placebo-controlled trial. Wound Repair Regen. (2019) 27:277–84. doi: 10.1111/wrr.12701, PMID: 30693609

[38] Uchitomi R Oyabu M Kamei Y. Vitamin D and sarcopenia: potential of vitamin D supplementation in sarcopenia prevention and treatment. Nutrients. (2020) 12. doi: 10.3390/nu12103189, PMID: 33086536 PMC7603112

[39] Kupisz-Urbańska M Płudowski P Marcinowska-Suchowierska E. Vitamin D deficiency in older patients-problems of sarcopenia, drug interactions, management in deficiency. Nutrients. (2021) 13. doi: 10.3390/nu13041247, PMID: 33920130 PMC8069639

[40] Chevalley T Brandi ML Cashman KD Cavalier E Harvey NC Maggi S . Role of vitamin D supplementation in the management of musculoskeletal diseases: update from an European Society of Clinical and Economical Aspects of osteoporosis, osteoarthritis and musculoskeletal diseases (ESCEO) working group. Aging Clin Exp Res. (2022) 34:2603–23. doi: 10.1007/s40520-022-02279-6, PMID: 36287325 PMC9607746

[41] Naweed SA Bess D-H Jason N David DA Anastassios GP. Vitamin D status of black and white Americans and changes in vitamin D metabolites after varied doses of vitamin D supplementation. Am J Clin Nutr. (2016) 104:205–14. doi: 10.3945/ajcn.115.129478, PMID: 27194308 PMC4919528

[42] Palacios C Kostiuk LK Peña-Rosas JP. Vitamin D supplementation for women during pregnancy. Cochrane Database Syst Rev. (2019) 2019:CD008873. doi: 10.1002/14651858.CD008873.pub4, PMID: 31348529 PMC6659840

[43] Kim KB Kim HW Lee JS Yoon SM. Inflammatory bowel disease and vitamin D. Korean J Gastroenterol. (2020) 76:275–81. doi: 10.4166/kjg.2020.160, PMID: 33361704 PMC12286300

[44] Macchi C Sirtori CR Corsini A Santos RD Watts GF Ruscica M. A new dawn for managing dyslipidemias: the era of rna-based therapies. Pharmacol Res. (2019) 150:104413. doi: 10.1016/j.phrs.2019.104413, PMID: 31449975

[45] Grant WB Lahore H McDonnell SL Baggerly CA French CB Aliano JL . Evidence that vitamin D supplementation could reduce risk of influenza and COVID-19 infections and deaths. Nutrients. (2020) 12. doi: 10.3390/nu12040988, PMID: 32252338 PMC7231123

[46] Jolliffe DA Camargo CA Jr Sluyter JD Aglipay M Aloia JF Ganmaa D . Vitamin D supplementation to prevent acute respiratory infections: a systematic review and meta-analysis of aggregate data from randomised controlled trials. Lancet Diabetes Endocrinol. (2021) 9:276–92. doi: 10.1016/s2213-8587(21)00051-6, PMID: 33798465

[47] Anna Maria R Szymon H Ryszard S Agnieszka D Iwona K-K. Antioxidant effects of vitamin E and risk of cardiovascular disease in women with obesity – a narrative review. Clin Nutr. (2022) 41:1557–65. doi: 10.1016/j.clnu.2022.04.032, PMID: 35667272

[48] Welma S Grant DB Campbell HT Mahinda YA. Short term effects of palm-tocotrienol and palm-carotenes on vascular function and cardiovascular disease risk: a randomised controlled trial. Atherosclerosis. (2016) 254:205–14. doi: 10.1016/j.atherosclerosis.2016.10.027, PMID: 27760402

[49] Meydani SN Barklund MP Liu S Meydani M Miller RA Cannon JG . Vitamin E supplementation enhances cell-mediated immunity in healthy elderly subjects. Am J Clin Nutr. (1990) 52:557–63. doi: 10.1093/ajcn/52.3.557, PMID: 2203257

[50] Han SN Wu D Ha WK Beharka A Smith DE Bender BS . Vitamin E supplementation increases T helper 1 cytokine production in old mice infected with influenza virus. Immunology. (2000) 100:487–93. doi: 10.1046/j.1365-2567.2000.00070.x, PMID: 10929076 PMC2327029

[51] Bou Ghanem EN Clark S Du X Wu D Camilli A Leong JM . The α-tocopherol form of vitamin E reverses age-associated susceptibility to *Streptococcus pneumoniae* lung infection by modulating pulmonary neutrophil recruitment. J Immunol. (2014) 194:1090–9. doi: 10.4049/jimmunol.1402401, PMID: 25512603 PMC4834212

[52] Bai Y Li T Liu J Wang Y Wang C Ju S . Aerobic exercise and vitamin E improve high-fat diet-induced NAFLD in rats by regulating the AMPK pathway and oxidative stress. Eur J Nutr. (2023) 62:2621–32. doi: 10.1007/s00394-023-03179-9, PMID: 37219594

[53] Moslehi N Vafa M Sarrafzadeh J Rahimi-Foroushani A. Does magnesium supplementation improve body composition and muscle strength in middle-aged overweight women? A double-blind, placebo-controlled, randomized clinical trial. Biol Trace Elem Res. (2013) 153:111–8. doi: 10.1007/s12011-013-9672-1, PMID: 23619906

[54] Veronese N Berton L Carraro S Bolzetta F De Rui M Perissinotto E . Effect of oral magnesium supplementation on physical performance in healthy elderly women involved in a weekly exercise program: a randomized controlled trial. Am J Clin Nutr. (2014) 100:974–81. doi: 10.3945/ajcn.113.080168 25008857

[55] Erem S Atfi A Razzaque MS. Anabolic effects of vitamin D and magnesium in aging bone. J Steroid Biochem Mol Biol. (2019) 193:105400. doi: 10.1016/j.jsbmb.2019.105400, PMID: 31175968

[56] Walker AF Marakis G Christie S Byng M. Mg citrate found more bioavailable than other Mg preparations in a randomised, double-blind study. Magnes Res. (2003) 16:183–91. 14596323

[57] Guerrera MP Volpe SL Mao JJ. Therapeutic uses of magnesium. Am Fam Physician. (2009) 80:157–62. 19621856

[58] Dong Y Yao L Cai L Jin M Forouzanfar T Wu L . Antimicrobial and pro-osteogenic coaxially electrospun magnesium oxide nanoparticles-polycaprolactone/parathyroid hormone-polycaprolactone composite barrier membrane for guided bone regeneration. Int J Nanomedicine. (2023) 18:369–83. doi: 10.2147/ijn.S395026, PMID: 36700148 PMC9869899

[59] Hanna T Mikko T Mirja P Juha O Sisko H Ritva V. Serum high-sensitive C-reactive protein may reflect periodontitis in patients with stroke. In Vivo. (2020) 34:2829–35. doi: 10.21873/invivo.12109, PMID: 32871821 PMC7652465

[60] Kim DJ Xun P Liu K Loria C Yokota K Jacobs DR Jr . Magnesium intake in relation to systemic inflammation, insulin resistance, and the incidence of diabetes. Diabetes Care. (2010) 33:2604–10. doi: 10.2337/dc10-0994, PMID: 20807870 PMC2992198

[61] Elena R-R Aránzazu A Pedro A Rosa MO. Moderate vitamin D deficiency and inflammation related markers in overweight/obese schoolchildren. Int J Vitam Nutr Res. (2014) 84:98–107. doi: 10.1024/0300-9831/a000197, PMID: 25835240

[62] Piscaer TM Müller C Mindt TL Lubberts E Verhaar JA Krenning EP . Imaging of activated macrophages in experimental osteoarthritis using folate-targeted animal single-photon-emission computed tomography/computed tomography. Arthritis Rheum. (2011) 63:1898–907. doi: 10.1002/art.30363, PMID: 21437875

[63] de Medeiros Cavalcante IG Silva AS Costa MJ Persuhn DC Issa CT de Luna Freire TL . Effect of vitamin D3 supplementation and influence of Bsm I polymorphism of the VDR gene of the inflammatory profile and oxidative stress in elderly women with vitamin D insufficiency: vitamin D3 megadose reduces inflammatory markers. Exp Gerontol. (2015) 66:10–6. doi: 10.1016/j.exger.2015.03.011 25827670

[64] Huang W Ma X Chen Y Zheng J Li H Nizhamu A . Dietary magnesium intake modifies the association between vitamin D and systolic blood pressure: results from NHANES 2007–2014. Front Nutr. (2022) 9:829857. doi: 10.3389/fnut.2022.829857, PMID: 35284447 PMC8908235

[65] Liu Y Gong R Ma H Chen S Sun J Qi J . Dietary magnesium intake level modifies the association between vitamin D and insulin resistance: a large cross-sectional analysis of American adults. Front Nutr. (2022) 9:878665. doi: 10.3389/fnut.2022.878665, PMID: 35747262 PMC9211020

[66] WNFH WN Zulkeflee HA Ab Rahim SN Tuan Ismail TS. Association of vitamin D and magnesium with insulin sensitivity and their influence on glycemic control. World J Diabetes. (2023) 14:26–34. doi: 10.4239/wjd.v14.i1.26, PMID: 36684386 PMC9850798

[67] AlShaibani T Abdul Razzaq R Radhi A Meer H Aljawder A Jaradat A . Ethnic-based assessment of vitamin D and magnesium status in the Kingdom of Bahrain. Cureus. (2024) 16:e55967. doi: 10.7759/cureus.55967, PMID: 38469368 PMC10927250

[68] Silva MC Furlanetto TW. Does serum 25-hydroxyvitamin D decrease during acute-phase response? A systematic review. Nutr Res. (2015) 35:91–6. doi: 10.1016/j.nutres.2014.12.008, PMID: 25631715

[69] Moslehi N Vafa M Rahimi-Foroushani A Golestan B. Effects of oral magnesium supplementation on inflammatory markers in middle-aged overweight women. J Res Med Sci. (2013) 17:607–14. PMC3685774 23798918

[70] Forrest HN. Magnesium, inflammation, and obesity in chronic disease. Nutr Rev. (2010) 68:333–40. doi: 10.1111/j.1753-4887.2010.00293.x, PMID: 20536778

[71] Hana Z Jan H. TNF-α signalling and inflammation: interactions between old acquaintances. Inflamm Res. (2013) 62:641–51. doi: 10.1007/s00011-013-0633-0, PMID: 23685857

[72] Paolo DG Cosimo Andrea S Paolo G Tannaz J Alexandra E B Amirhossein S. The role of nutrition in inflammaging. Ageing Res Rev. (2022) 77:101596. doi: 10.1016/j.arr.2022.101596 35219904

[73] Weglicki WB Phillips TM Freedman AM Cassidy MM Dickens BF. Magnesium-deficiency elevates circulating levels of inflammatory cytokines and endothelin. Mol Cell Biochem. (1992) 110:169–73. doi: 10.1007/bf02454195, PMID: 1584207

[74] Schröder-Heurich B von Hardenberg S Brodowski L Kipke B Meyer N Borns K . Vitamin D improves endothelial barrier integrity and counteracts inflammatory effects on endothelial progenitor cells. FASEB J. (2019) 33:9142–53. doi: 10.1096/fj.201802750RR, PMID: 31084577

[75] Maryam E Amir Hossein R Sara Soleimani A Saed B Effat Sadat MM Fereshteh M. The effects of resveratrol on silica-induced lung oxidative stress and inflammation in rat. *Saf health*. Work. (2023) 14:118–23. doi: 10.1016/j.shaw.2023.02.001, PMID: 36941929 PMC10024237

[76] Saboori S Shab-Bidar S Speakman JR Yousefi Rad E Djafarian K. Effect of vitamin E supplementation on serum C-reactive protein level: a meta-analysis of randomized controlled trials. Eur J Clin Nutr. (2015) 69:867–73. doi: 10.1038/ejcn.2014.296, PMID: 25669317

[77] Mazur A Maier J Rock E Gueux E Nowacki W Rayssiguier Y. Magnesium and the inflammatory response: potential physiopathological implications. Arch Biochem Biophys. (2007) 458:48–56. doi: 10.1016/j.abb.2006.03.031, PMID: 16712775

[78] Kim D Kim J Ham H Choue R. Effects of d-α-tocopherol supplements on lipid metabolism in a high-fat diet-fed animal model. Nutr Res Pract. (2013) 7:481–7. doi: 10.4162/nrp.2013.7.6.481, PMID: 24353834 PMC3865271

[79] Kishimoto Y Tani M Uto-Kondo H Saita E Iizuka M Sone H . Effects of magnesium on postprandial serum lipid responses in healthy human subjects. Br J Nutr. (2010) 103:469–72. doi: 10.1017/s0007114509992716 19941679

[80] Guerrero-Romero F Simental-Mendía LE Hernández-Ronquillo G Rodriguez-Morán M. Oral magnesium supplementation improves glycaemic status in subjects with prediabetes and hypomagnesaemia: a double-blind placebo-controlled randomized trial. Diabetes Metab. (2015) 41:202–7. doi: 10.1016/j.diabet.2015.03.010, PMID: 25937055

[81] Ekhlasi G Kolahdouz Mohammadi R Agah S Zarrati M Hosseini AF Arabshahi SS . Do symbiotic and vitamin E supplementation have favorite effects in nonalcoholic fatty liver disease? A randomized, double-blind, placebo-controlled trial. J Res Med Sci. (2017) 21:106. doi: 10.4103/1735-1995.193178, PMID: 28250783 PMC5322689

[82] Xu R Zhang S Tao A Chen G Zhang M. Influence of vitamin E supplementation on glycaemic control: a meta-analysis of randomised controlled trials. PLoS One. (2014) 9:e95008. doi: 10.1371/journal.pone.0095008, PMID: 24740143 PMC3989270

[83] Simental-Mendía LE Simental-Mendía M Sahebkar A Rodríguez-Morán M Guerrero-Romero F. Effect of magnesium supplementation on lipid profile: a systematic review and meta-analysis of randomized controlled trials. Eur J Clin Pharmacol. (2017) 73:525–36. doi: 10.1007/s00228-017-2212-8, PMID: 28180945

[84] Dou M Ma AG Wang QZ Liang H Li Y Yi XM . Supplementation with magnesium and vitamin E were more effective than magnesium alone to decrease plasma lipids and blood viscosity in diabetic rats. Nutr Res. (2009) 29:519–24. doi: 10.1016/j.nutres.2009.07.001, PMID: 19700040

[85] Tao Y Pan Y Wang Q Lu S Li Y Liu W . Vitamin E ameliorates impaired ovarian development, oxidative stress, and disrupted lipid metabolism in *Oreochromis niloticus* fed with a diet containing olive oil instead of fish oil. Antioxidants (Basel). (2023) 12. doi: 10.3390/antiox12081524, PMID: 37627518 PMC10451663

[86] Al-Okbi SY El-Qousy SM El-Ghlban S Moawad HF. Role of borage seed oil and fish oil with or without turmeric and alpha-tocopherol in prevention of cardiovascular disease and fatty liver in rats. J Oleo Sci. (2018) 67:1551–62. doi: 10.5650/jos.ess18064, PMID: 30429440

[87] Ostadmohammadi V Samimi M Mobini M Zarezade Mehrizi M Aghadavod E Chamani M . The effect of zinc and vitamin E cosupplementation on metabolic status and its related gene expression in patients with gestational diabetes. J Matern Fetal Neonatal Med. (2018) 32:4120–7. doi: 10.1080/14767058.2018.1481952, PMID: 29804469

