# Supplementary material for: The effects of magnesium and vitamin D/E co-supplementation on inflammation markers and lipid metabolism of obese/overweight population: a systematic review and meta-analysis
Source: Front Nutr. 2025 Sep 1;12:1563604. doi: 10.3389/fnut.2025.1563604 (PMC12433974; doi:10.3389/fnut.2025.1563604)
Supplement: Supplementary file 1 [file Table_1.docx]

| Database name | Search strategies: key words and how these were combined in the search | Retrieval time | Number of studies identified |
| --- | --- | --- | --- |
| PubMed | （ co-supplementation OR combined supplementation）AND(((("Vitamin E"[Mesh]) OR ("Vitamin D"[Mesh])) AND ("Magnesium"[Mesh])) AND (((("Oxidative Stress"[Mesh]) OR (((((((((((((((((((((((((((((((((((((((((Oxidative Stresses) ) OR (Stress, Oxidative)) OR (Oxidative DNA Damage)) OR (Damage, Oxidative DNA)) OR (DNA Damage, Oxidative)) OR (Oxidative DNA Damages)) OR (DNA Oxidative Damage)) OR (Damage, DNA Oxidative)) OR (DNA Oxidative Damages)) OR (Oxidative Damage, DNA)) OR (Oxidative and Nitrosative Stress)) OR (Nitro-Oxidative Stress)) OR (Nitro Oxidative Stress)) OR (Nitro-Oxidative Stresses)) OR (Stresses, Nitro-Oxidative)) OR (Stress, Nitro-Oxidative)) OR (Oxidative Nitrative Stress)) OR (Nitrative Stress, Oxidative)) OR (Oxidative Nitrative Stresses)) OR (Stress, Oxidative Nitrative)) OR (Oxidative Damage)) OR (Damage, Oxidative)) OR (Oxidative Damages)) OR (Oxidative Injury)) OR (Injury, Oxidative)) OR (Oxidative Injuries)) OR (Oxidative Stress Injury)) OR (Injury, Oxidative Stress)) OR (Oxidative Stress Injuries)) OR (Stress Injury, Oxidative)) OR (Oxidative Cleavage)) OR (Cleavage, Oxidative)) OR (Oxidative Cleavages)) OR (Antioxidative Stress)) OR (Antioxidative Stresses)) OR (Stress, Antioxidative)) OR (Anti-oxidative Stress)) OR (Anti oxidative Stress)) OR (Anti-oxidative Stresses)) OR (Stress, Anti-oxidative))) OR (("Inflammation"[Mesh]) OR ((((Inflammations) OR (Innate Inflammatory Response)) OR (Inflammatory Response, Innate)) OR (Innate Inflammatory Responses)))) OR (("Metabolism"[Mesh]) OR ((((((((((((((((((((Metabolic Process) OR (Process, Metabolic)) OR (Processes, Metabolic)) OR (Metabolic Processes)) OR (Anabolism)) OR (Catabolism)) OR (Metabolic Phenomena)) OR (Phenomena, Metabolic)) OR (Metabolic Concepts)) OR (Concept, Metabolic)) OR (Concepts, Metabolic)) OR (Metabolic Concept)) OR (Metabolism Concepts)) OR (Concept, Metabolism)) OR (Concepts, Metabolism)) OR (Metabolism Concept)) OR (Metabolism Phenomena)) OR (Phenomena, Metabolism)) OR (Metabolic Phenomenon)) OR (Phenomenon, Metabolic))))) | January  2024 | 32 |
| Web of science | #1TS=((co-supplementation) OR (combined supplementation))  #2TS=(Magnesium)  #3TS=((Vitamin D)  #4TS=(Vitamin E)  #5 #3 OR #4  #6TS=((Oxidative stress) OR ( Oxidative Stresses) OR (Cleavage, Oxidative) OR (Oxidative Cleavages) OR (Antioxidative Stress) OR (Antioxidative Stresses) OR (Stress, Antioxidative) OR (Anti-oxidative Stress ) OR (Anti oxidative Stress) OR ( Anti-oxidative Stresses) OR ( Stress, Anti-oxidative) OR (Stress Oxidative) OR ( Oxidative DNA Damage) OR ( Damage, Oxidative DNA ) OR (DNA Damage, Oxidative) OR ( Oxidative DNA Damages) OR ( DNA Oxidative Damage ) OR (Damage, DNA Oxidative) OR ( DNA Oxidative Damages) OR ( Oxidative Damage, DNA) OR (Oxidative and Nitrosative Stress ) OR (Nitro-Oxidative Stress ) OR (Nitro Oxidative Stress) OR ( Nitro-Oxidative Stresses) OR ( Stresses, Nitro-Oxidative) OR ( Stress, Nitro-Oxidative) OR ( Oxidative Nitrative Stress ) OR (Nitrative Stress, Oxidative) OR ( Oxidative Nitrative Stresses) OR ( Stress, Oxidative Nitrative) OR ( Oxidative Damage ) OR (Damage, Oxidative ) OR (Oxidative Damages) OR ( Oxidative Injury) OR ( Injury, Oxidative) OR ( Oxidative Injuries) OR (Oxidative Stress Injury ) OR (Injury, Oxidative Stress) OR ( Oxidative Stress Injuries ) OR (Stress Injury, Oxidative ) OR (Oxidative Cleavage))  #7TS=((Inflammation) OR (Inflammations) OR (Innate Inflammatory Response) OR ( Inflammatory Response, Innate) OR (Innate Inflammatory Responses))  #8TS=((Metabolism) OR (Metabolic Process) OR (Process, Metabolic) OR (Processes, Metabolic) OR (Metabolic Processes) OR (Anabolism) OR (Catabolism) OR (Metabolic Phenomena) OR (Phenomena, Metabolic) OR (Metabolic Concepts) OR (Concept, Metabolic) OR (Concepts, Metabolic) OR ( Metabolic Concept) OR ( Metabolism Concepts) OR ( Concept, Metabolism) OR ( Concepts, Metabolism ) OR (Metabolism Concept) OR ( Metabolism Phenomena) OR ( Phenomena, Metabolism ) OR (Metabolic Phenomenon) OR ( Phenomenon, Metabolic))  #9 #6 OR #7 OR #8  #1 AND #2 AND #5 AND #9 | January  2024 | 91 |
| The Cochrane library | #1 MeSH descriptor: [Magnesium] explode all trees  #2 MeSH descriptor: [Vitamin D] explode all trees  #3 MeSH descriptor: [Vitamin E] explode all trees  #4 #2 OR #3  #5 MeSH descriptor: [Oxidative Stress] explode all trees  #6(Oxidative stress):ti,ab,kw OR ( Oxidative Stresses):ti,ab,kw OR (Cleavage, Oxidative):ti,ab,kw OR (Oxidative Cleavages):ti,ab,kw OR (Antioxidative Stress):ti,ab,kw OR (Antioxidative Stresses):ti,ab,kw OR (Stress, Antioxidative):ti,ab,kw OR (Anti-oxidative Stress ):ti,ab,kw OR (Anti oxidative Stress):ti,ab,kw OR ( Anti-oxidative Stresses):ti,ab,kw OR ( Stress, Anti-oxidative):ti,ab,kw OR (Stress Oxidative):ti,ab,kw OR ( Oxidative DNA Damage):ti,ab,kw OR ( Damage, Oxidative DNA ):ti,ab,kw OR (DNA Damage, Oxidative):ti,ab,kw OR ( Oxidative DNA Damages):ti,ab,kw OR ( DNA Oxidative Damage ):ti,ab,kw OR (Damage, DNA Oxidative):ti,ab,kw OR ( DNA Oxidative Damages):ti,ab,kw OR ( Oxidative Damage, DNA):ti,ab,kw OR (Oxidative and Nitrosative Stress ):ti,ab,kw OR (Nitro-Oxidative Stress ):ti,ab,kw OR (Nitro Oxidative Stress):ti,ab,kw OR ( Nitro-Oxidative Stresses):ti,ab,kw OR ( Stresses, Nitro-Oxidative):ti,ab,kw OR ( Stress, Nitro-Oxidative):ti,ab,kw OR ( Oxidative Nitrative Stress ):ti,ab,kw OR (Nitrative Stress, Oxidative):ti,ab,kw OR ( Oxidative Nitrative Stresses):ti,ab,kw OR ( Stress, Oxidative Nitrative):ti,ab,kw OR ( Oxidative Damage ):ti,ab,kw OR (Damage, Oxidative ):ti,ab,kw OR (Oxidative Damages):ti,ab,kw OR ( Oxidative Injury):ti,ab,kw OR ( Injury, Oxidative):ti,ab,kw OR ( Oxidative Injuries):ti,ab,kw OR (Oxidative Stress Injury ):ti,ab,kw OR (Injury, Oxidative Stress):ti,ab,kw OR ( Oxidative Stress Injuries ):ti,ab,kw OR (Stress Injury, Oxidative ):ti,ab,kw OR (Oxidative Cleavage):ti,ab,kw  #7 MeSH descriptor: [Inflammation] explode all trees  #8 (Inflammation):ti,ab,kw OR (Inflammations):ti,ab,kw OR (Innate Inflammatory Response):ti,ab,kw OR ( Inflammatory Response, Innate):ti,ab,kw OR (Innate Inflammatory Responses):ti,ab,kw  #9 MeSH descriptor: [Metabolism] explode all trees  #10(Metabolism):ti,ab,kw OR (Metabolic Process):ti,ab,kw OR (Process, Metabolic):ti,ab,kw OR (Processes, Metabolic):ti,ab,kw OR (Metabolic Processes):ti,ab,kw OR (Anabolism):ti,ab,kw OR (Catabolism):ti,ab,kw OR (Metabolic Phenomena):ti,ab,kw OR (Phenomena, Metabolic):ti,ab,kw OR (Metabolic Concepts):ti,ab,kw OR (Concept, Metabolic):ti,ab,kw OR (Concepts, Metabolic):ti,ab,kw OR ( Metabolic Concept):ti,ab,kw OR ( Metabolism Concepts):ti,ab,kw OR ( Concept, Metabolism):ti,ab,kw OR ( Concepts, Metabolism ):ti,ab,kw OR (Metabolism Concept):ti,ab,kw OR ( Metabolism Phenomena):ti,ab,kw OR ( Phenomena, Metabolism ):ti,ab,kw OR (Metabolic Phenomenon):ti,ab,kw OR (Phenomenon, Metabolic):ti,ab,kw  #11 #5 OR #6 OR #7 OR #8 OR #9 OR #10  #12 MeSH descriptor: [Metabolism] explode all trees  #13 (co-supplementaion):ti,ab,kw OR (combined supplementation):ti,ab,kw  #14 #12 OR #13  #15 #1 AND #4 AND #11 AND #14 | January  2024 | 9 |
| Embase | #1 'Magnesium'  #2 'Vitamin D'  #3 'Vitamin E'  #4 #2 OR #3  #5'Co-supplementation' OR 'combined supplementation' #6'Oxidative stress' OR ' Oxidative Stresses ' OR 'Cleavage, Oxidative' OR 'Oxidative Cleavages' OR 'Antioxidative Stress' OR 'Antioxidative Stresses' OR 'Stress, Antioxidative' OR 'Anti-oxidative Stress ' OR 'Anti oxidative Stress' OR ' Anti-oxidative Stresses' OR ' Stress, Anti-oxidative' OR 'Stress Oxidative' OR ' Oxidative DNA Damage' OR ' Damage, Oxidative DNA ' OR 'DNA Damage, Oxidative' OR ' Oxidative DNA Damages' OR ' DNA Oxidative Damage ' OR 'Damage, DNA Oxidative' OR ' DNA Oxidative Damages' OR ' Oxidative Damage, DNA' OR 'Oxidative and Nitrosative Stress ' OR 'Nitro-Oxidative Stress ' OR 'Nitro Oxidative Stress' OR ' Nitro-Oxidative Stresses' OR ' Stresses, Nitro-Oxidative' OR ' Stress, Nitro-Oxidative' OR ' Oxidative Nitrative Stress ' OR 'Nitrative Stress, Oxidative' OR ' Oxidative Nitrative Stresses' OR ' Stress, Oxidative Nitrative' OR ' Oxidative Damage ' OR 'Damage, Oxidative ' OR 'Oxidative Damages' OR ' Oxidative Injury' OR ' Injury, Oxidative' OR ' Oxidative Injuries' OR 'Oxidative Stress Injury ' OR 'Injury, Oxidative Stress' OR ' Oxidative Stress Injuries ' OR 'Stress Injury, Oxidative ' OR 'Oxidative Cleavage' #7'Inflammation' OR 'Innate Inflammatory Response' OR ' Inflammatory Response, Innate' OR 'Innate Inflammatory Responses' OR ' Inflammations'  #8'Metabolism' OR 'Metabolic Process' OR 'Process, Metabolic' OR 'Processes, Metabolic' OR 'Metabolic Processes' OR 'Anabolism' OR 'Catabolism' OR 'Metabolic Phenomena' OR 'Phenomena, Metabolic' OR 'Metabolic Concepts' OR 'Concept, Metabolic' OR 'Concepts, Metabolic' OR ' Metabolic Concept' OR ' Metabolism Concepts' OR ' Concept, Metabolism' OR ' Concepts, Metabolism ' OR 'Metabolism Concept' OR ' Metabolism Phenomena' OR ' Phenomena, Metabolism ' OR 'Metabolic Phenomenon' OR 'Phenomenon, Metabolic'  #9 #6 OR #7 OR #8 #1 AND #4 AND #5 AND #9 | January  2024 | 21 |

Supplementary table 1 Retrieval formula


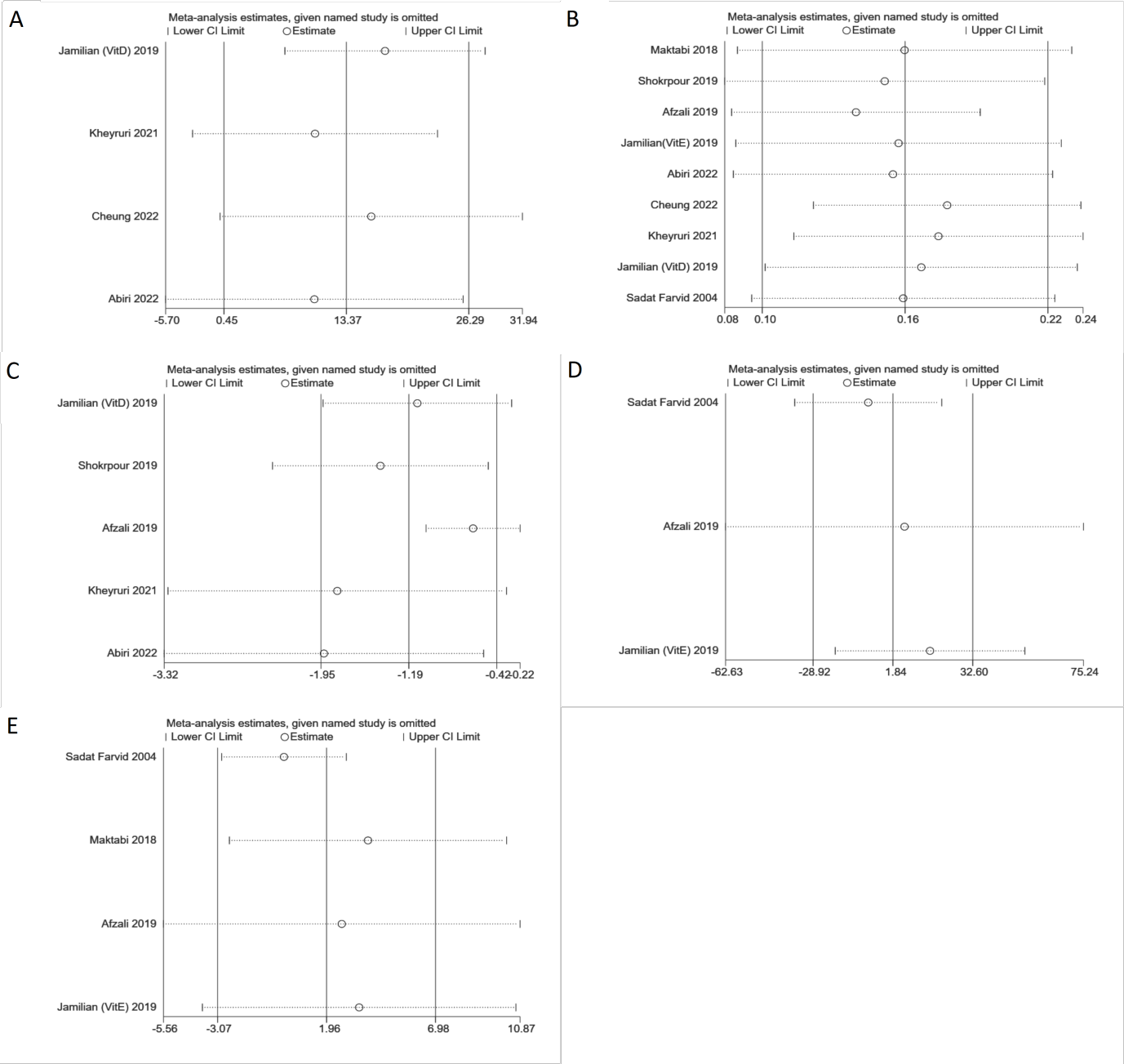


Supplementary table 2 Sensitivity analyses

A: Sensitivity analysis of magnesium and vitamin D co-supplementation effects on 25(OH)D.

B: Sensitivity analysis of magnesium and vitamin D/E co-supplementation effects on magnesium.

C: Sensitivity analysis of magnesium and vitamin D co- supplementation effects on hs-CRP.

D:Sensitivity analysis of magnesium and vitamin E co- supplementation effects on triglyceride.

E:Sensitivity analysis of magnesium and vitamin E co-supplementation effects on high density lipoprotein cholesterol.

| Outcomes | Std_Eff | Coef. | Std. Err | t | P | 95%CI |
| --- | --- | --- | --- | --- | --- | --- |
| 25(OH)D | slope | 15.71392 | 21.0231 | 0.75 | 0.533 | -74.74117 to 106.169 |
|  | bias | -1.764746 | 16.92032 | -0.10 | 0.926 | -74.56699 to 71.0375 |
| Mg | slope | 0.194266 | 0.1475036 | 1.32 | 0.229 | -0.1545245 to 0.5430565 |
|  | bias | -0.7641003 | 3.343974 | -0.23 | 0.826 | -8.671342 to 7.143142 |
| Hs-CRP | slope | 0.0072727 | 0.2928877 | 0.02 | 0.982 | -0.9248265 to 0.939372 |
|  | bias | -3.335428 | 1.425985 | -2.34 | 0.101 | -7.873548 to 1.202692 |
| IL-6 | slope | 0.0232345 | 0.4435532 | 0.05 | 0.967 | -5.612643 to 5.659112 |
|  | bias | -0.2994216 | 2.111061 | -0.14 | 0.910 | -27.12299 to 26.52415 |
| TG | slope | -19.11127 | 50.35926 | -0.38 | 0.769 | -658.9863 to 620.7638 |
|  | bias | 1.33706 | 3.359208 | 0.40 | 0.759 | -41.34572 to 44.01984 |
| LDL-c | slope | 29.23208 | 95.81378 | 0.31 | 0.789 | -383.0214 to 441.4855 |
|  | bias | -3.447676 | 9.747495 | -0.35 | 0.757 | -45.38776 to 38.49241 |
| HDL-c | slope | -4.649392 | 7.495053 | -0.62 | 0.598 | -36.898 to 27.59922 |
|  | bias | 2.509611 | 3.102548 | 0.81 | 0.504 | -10.83957 to 15.8588 |

Supplementary table 3 Egger’s test results for the meta-analysis.
